# Atrial Fibrillation in Infiltrative Cardiomyopathies: From Atrial Cardiomyopathy Imaging to Targeted Management—A Narrative Review

**DOI:** 10.3390/medicina62061023

**Published:** 2026-05-25

**Authors:** Matteo Brusamolino, Francesco Catagnano, Maria Lo Monaco, Silvia Malara, Matteo De Carli, Margherita Licastro, Valentina Rossi, Gaspare Maranzano, Federica Frascaro, Stefano Frittella, Flavia Nicoli, Rocco Mollace, Erika Bertella

**Affiliations:** 1 Department of Cardiology, Humanitas Gavazzeni Hospital, Via Gavazzeni 21, 24125 Bergamo, Italy; 2 Department of Biomedical Sciences, Humanitas University, Pieve Emanuele, 20072 Milan, Italy

**Keywords:** atrial fibrillation, infiltrative cardiomyopathies, atrial cardiomyopathy

## Abstract

Infiltrative cardiomyopathies comprise a heterogeneous spectrum of hereditary and acquired diseases characterised by the accumulation of pathological substrates within the myocardium, ultimately resulting in progressive impairment of cardiac function and the development of heart failure. Across these conditions, atrial fibrillation is a frequent and clinically relevant complication, contributing to symptom burden, heart failure progression, and thromboembolic risk. Structural, functional, and electrical atrial remodelling, collectively referred to as atrial cardiomyopathy, emerges as a common pathophysiological substrate linking myocardial infiltration to atrial fibrillation and adverse cardiovascular outcomes. Multi-modality cardiac imaging enables comprehensive assessment of atrial cardiomyopathy, offering mechanistic insights into the atrial substrate of atrial fibrillation, with potential impact on the clinical management of this group of diseases. This review summarises contemporary evidence on atrial fibrillation in infiltrative cardiomyopathies, with a particular focus on the role of non-invasive multimodal imaging in the evaluation of atrial cardiomyopathy.

## 1. Introduction

Infiltrative cardiomyopathies (Inf-CM) encompass a heterogeneous group of inherited and acquired disorders characterised by myocardial deposition of abnormal substances, either within the extracellular matrix or intracellular compartment [1]. The most prevalent forms include cardiac amyloidosis (CA), Anderson–Fabry disease (FD), cardiac sarcoidosis (CS) and iron overload cardiomyopathy (IOC). Myocardial infiltration disrupts normal cardiac structure and function, promotes inflammation and fibrosis, leading to diastolic dysfunction, systolic impairment or both, and ultimately to heart failure and increased mortality.

Atrial fibrillation (AF) represents a frequent and clinically relevant complication across this disease spectrum. In affected patients, the atria commonly exhibit a constellation of structural remodelling, functional impairment, and electrical abnormalities. These alterations are collectively encompassed by the concept of atrial cardiomyopathy (A-CM), which has been proposed as a unifying pathological substrate underlying adverse cardiovascular outcomes, including heart failure progression, AF and thromboembolic events [2]. Because clinical expertise and evidence regarding right atrial size, function and remodelling are still developing, this review focuses primarily on the comprehensive assessment of LA cardiomyopathy [3]. Multimodality cardiac imaging offers a comprehensive, non-invasive approach for the detailed characterisation of A-CM. Transthoracic echocardiography (TTE) is the first-line imaging modality owing to its wide availability and low cost, enabling evaluation of atrial size, phasic function, and deformation parameters, with strain imaging providing sensitive markers of early atrial dysfunction. Transesophageal echocardiography (TEE) provides superior spatial resolution for the evaluation of left atrial appendage (LAA) anatomy and function due to its close anatomic proximity to the esophagus, and remains the reference technique for detection and exclusion of atrial thrombus and spontaneous echo contrast. Of note, quantitative assessment of atrial dimensions is not usually performed using TEE, as its imaging sector does not reliably encompass the entire atrial chamber. Cardiovascular magnetic resonance (CMR) enables comprehensive tissue characterisation, accurate atrial anatomic, volumetric and functional assessments, and detection of atrial fibrosis through late gadolinium enhancement (LGE). Cardiac computed tomography (CCT) offers high-resolution three-dimensional visualisation of atrial anatomy, precise volumetric quantification, and detailed assessment of the LAA. Finally, scintigraphy and positron emission tomography (PET) have a role in the diagnostic workup of specific Inf-CM, including CA and CS. By integrating morphological, functional, and tissue characterisation data, advanced imaging techniques offer important mechanistic insights into the atrial substrate predisposing to AF. It has the potential to support diagnosis, risk stratification and therapeutic decision-making.

In this narrative review, we synthesise the most recent evidence on AF in Inf-CM, with particular emphasis on the contribution of multi-modality imaging to the assessment of A-CM in this group of diseases. We acknowledge that CA currently provides the most robust body of evidence in the context of A-CM; accordingly, the sections addressing other infiltrative and storage disorders are intentionally more concise, reflecting the relative paucity and heterogeneity of available data.

## 2. Search Strategy, Study Selection and Review Design

A literature search was conducted in PubMed/MEDLINE and Google Scholar for articles published from inception through 31 March 2026, using keywords related to atrial cardiomyopathy, infiltrative diseases, and multimodal imaging. We prioritised high-impact original research and international consensus statements, with a preference for studies published in the last decade to reflect contemporary imaging advancements. In cases of overlapping evidence or cohorts, the most comprehensive or recent data were selected. This manuscript is a narrative synthesis and was not conducted according to a predefined systematic protocol. Consequently, study selection was not performed independently in duplicate, and no formal risk-of-bias assessment was conducted. The review provides a descriptive expert overview, emphasising clinical relevance and the integration of multimodal imaging into current diagnostic paradigms for infiltrative cardiomyopathies.

## 3. A-CM in Inf-CM and the Role of Multimodal Imaging in LA Assessment

Research in Inf-CM has predominantly focused on ventricular involvement, whereas substantially less attention has been given to LA pathology, whose dilation and dysfunction have traditionally been regarded as secondary consequences of the elevated left ventricular (LV) filling pressures characteristic of this group of disorders. However, histopathological analyses and multimodal imaging studies strongly support that these diseases lead to an intrinsic (primary) LA cardiopathy, driven directly by atrial infiltration, with subsequent structural remodelling and loss of physiological function, that cannot be explained solely by a secondary consequence of ventricular dysfunction [4,5,6,7,8,9,10,11]. Consequently, the concept of A-CM has emerged as a clinically relevant entity, encompassing a complex and heterogeneous spectrum of structural, functional, and electrophysiological alterations of the atrial myocardium that predispose patients to adverse outcomes, including AF, stroke, and heart failure [2]. Importantly, current evidence suggests a bidirectional relationship between A-CM and AF: A-CM independently predicts the onset of AF, whereas AF is believed to contribute to the progression of A-CM [12]. Although this paradigm is most convincingly supported in CA, where the evidence base is extensive and consistent, data in other Inf-CM remain comparatively limited. Nonetheless, emerging observations suggest that A-CM may represent a relevant pathophysiological mechanism across these conditions. Despite its strong conceptual relevance, diagnosing A-CM remains challenging [2]. A recent consensus statement issued by the Heart Failure Association (HFA) of the European Society of Cardiology (ESC) has outlined an updated clinical framework for A-CM, introducing revised definitions together with refined diagnostic criteria and disease staging [13].

The comprehensive characterisation of the abnormalities associated with A-CM requires the rational use of multimodal imaging, with careful consideration of each technique’s strengths and limitations to yield information of clinical relevance [3,14]. A comparison of the main atrial-specific findings in Inf-CM, and the strengths and limitations of each non-invasive imaging modality for LA assessment in Inf-CM is proposed in Table 1.

A more comprehensive characterization of A-CM has the potential to enhance our understanding of the atrial remodeling processes underlying AF and contributing to heart failure pathophysiology. Although not yet part of routine clinical practice, such advances may facilitate the identification of patients at increased risk of AF, support more tailored rhythm monitoring strategies, inform clinical decision-making, and help stratify the risk of adverse outcomes.

## 4. Cardiac Amyloidosis

### 4.1. Prevalence and Clinical Impact of AF in CA

CA is a severe and progressive Inf-CM characterised by extracellular accumulation of misfolded amyloid fibrils within the myocardial tissue. The disease arises either from rare pathogenic genetic variants in hereditary forms or from acquired conditions leading to amyloid formation [15]. Although traditionally regarded as uncommon, accumulating evidence indicates that CA is substantially underrecognized and represents an important, often overlooked cause of prevalent cardiac diseases and clinical syndromes [16]. AF is one of the most common arrhythmias observed in patients with CA [17,18]. Its development reflects advanced atrial structural and functional remodelling driven by amyloid deposition and systemic disease burden, with important implications for symptoms, hemodynamics and clinical management. Prevalence and incidence of AF in CA populations appear to vary substantially across studies. Prior literature has reported AF prevalence ranging approximately from 40% to 88% in transthyretin amyloid cardiomyopathy (ATTR-CM) [19,20,21,22,23] and from 9% to 28% in light-chain cardiac amyloidosis (AL-CA) [22,24,25]. Wild-type ATTR appears to be more strongly associated with AF, potentially reflecting the combined impact of advanced age, progressive atrial remodeling, and coexisting cardiovascular comorbidities [26]. The observed heterogeneity in AF prevalence and incidence is likely multifactorial and, at least in part, attributable to differences in study design, including cohort selection, duration of follow-up, and AF ascertainment methods (routine ECG recordings, ECG-Holter monitoring, symptom-driven evaluation or systematic detection through implantable monitoring devices) [19,23]. Clinically, AF identifies a more advanced CA phenotype. Patients with AF consistently exhibit worse New York Heart Association (NYHA) functional class, greater comorbidity burden and more pronounced hemodynamic impairment, including larger LA volumes, elevated pulmonary artery pressures and impaired right ventricular function. These features translate into higher rates of heart failure hospitalisation and increased health-care utilization [17,23].

The prognostic significance of AF with respect to all-cause mortality remains heterogeneous. While some observational studies report an association between AF and increased mortality, this relationship is frequently attenuated after adjustment for disease stage and markers of cardiac involvement, suggesting that AF primarily reflects disease severity rather than acting as an independent prognostic determinant, as demonstrated by recent analyses [17,23]. Accordingly, AF is increasingly regarded as a clinical marker of advanced CA rather than a standalone driver of adverse outcomes.

From a pathophysiological perspective, AF is often poorly tolerated in CA. The restrictive ventricular physiology characteristic of amyloid cardiomyopathy results in marked dependence on atrial contraction to maintain adequate ventricular filling. Consequently, loss of atrial systole combined with an irregular ventricular response may precipitate acute hemodynamic deterioration, hypotension and worsening congestion, frequently leading to urgent hospitalization [18,26,27]. Moreover, patients with CA exhibit a heightened propensity for intracardiac thrombus formation that appears disproportionate to conventional risk stratification. AF therefore substantially contributes to thromboembolic risk in this population, supporting a low threshold for anticoagulation irrespective of traditional scores [15,28].

### 4.2. Predictors and Risk Factors for AF in CA

Beyond traditional cardiovascular risk factors and comorbidities, several CA-specific clinical, structural, and electro-functional markers have been linked to both prevalent and incident AF. In CA, A-CM is driven by deposition of insoluble amyloid fibrils within atrial walls together with the hemodynamic consequences of ventricular impairment, which leads to the destruction of the native tissue architecture, vascular remodeling, capillary damage, and subendocardial fibrosis [4]. The resulting LA electrical, structural, and functional remodeling leads to conduction slowing and increased electrical heterogeneity, creating a substrate that favors re-entry and ectopic activity and substantially lowers the threshold for AF initiation and maintenance [26]. In this context, AF emerges as a dynamic manifestation of progressive amyloid-related atrial cardiomyopathy [23]. In mixed AL/ATTR cohorts, ATTR subtype confers a significantly higher risk of AF compared with AL, independent of age and other covariates [22]. Higher body mass index, impaired renal function and more advanced disease stage further increase AF risk. In ATTR-CM–specific cohorts, older age, advanced ATTR stage and LA enlargement consistently predict AF occurrence [17,23,26]. More recent studies incorporating markers of atrial electromechanical dysfunction have shown that prolonged total atrial conduction time and reduced left atrial ejection fraction (LAEF) independently predict new-onset AF, demonstrating independent prognostic utility superior to that of traditional clinical variables and echocardiographic parameters [29,30]. Taken together, these findings suggest that both traditional risk factors and disease-specific features (encompassing clinical characteristics, amyloid subtype, disease stage, structural remodeling, and markers of atrial electromechanical dysfunction) may collectively contribute to AF susceptibility in CA, although their relative weight and integration into risk stratification frameworks remain to be fully established.

### 4.3. LA Multimodality Imaging in CA

#### 4.3.1. TTE

**Figure 1:**
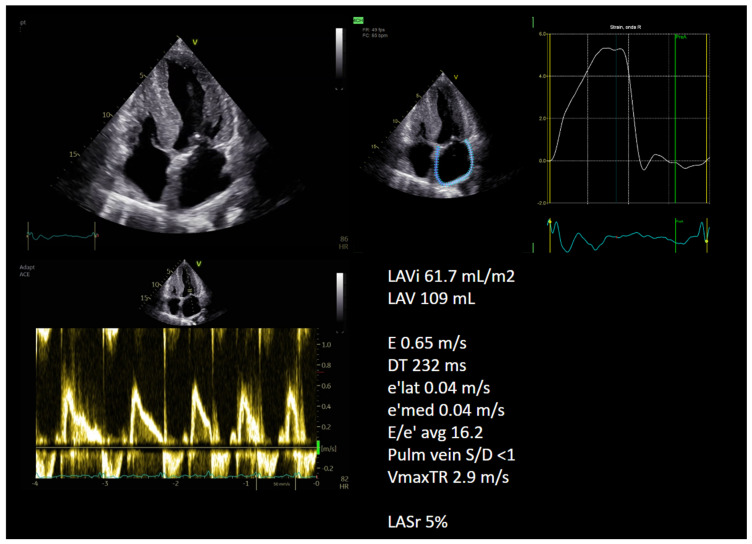
Typical echocardiographic features of a patient with transthyretin cardiac amyloidosis and atrial fibrillation: severe LA dilation and atrial wall thickening; markedly reduced left atrial reservoir strain, with absence of atrial contraction consistent with atrial fibrillation; diastolic dysfunction with indirect evidence of elevated left ventricular filling pressures. LAVi, left atrial volume indexed; LAV, left atrial volume; E, E wave; DT, deceleration time; E/e’avg, average E/e’; Pulm vein S/D, pulmonary vein systolic/diastolic ratio; VmaxTR, maximum tricuspid regurgitation velocity; LASr, Left Atrial Reservoir Strain.

**Figure 2:**
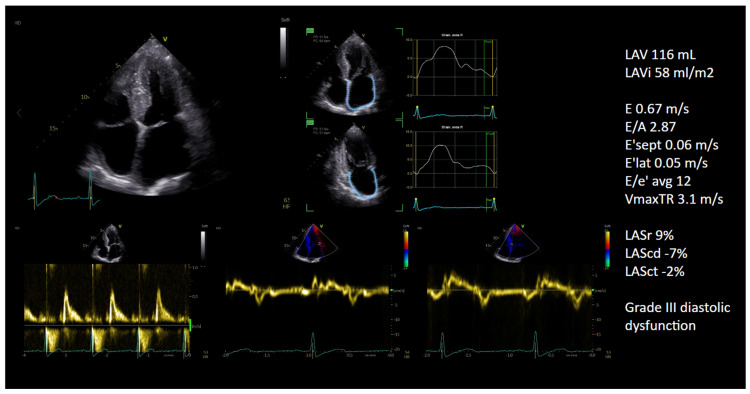
Typical echocardiographic features of a patient with transthyretin cardiac amyloidosis in sinus rhythm: severe left atrial dilation and atrial wall thickening; markedly reduced left atrium reservoir strain and contractile strain; diastolic dysfunction with indirect evidence of elevated left ventricular filling pressures. A subset of patients with cardiac amyloidosis and sinus rhythm may present atrial electromechanical dissociation (absent LASct and A wave, despite ECG evidence of sinus rhythm). LAVi, left atrial volume indexed; LAV, left atrial volume; E, E wave; A, A wave; E/e’avg, average E/e’; VmaxTR, maximum tricuspid regurgitation velocity; LASr, Left Atrial Reservoir Strain; LAScd, Left Atrial Conduit Strain.; LASct, Left Atrial Contractile Strain.

As previously discussed, TTE represents the first-line imaging modality for the assessment of LA size and function (Figure 1 and Figure 2). Increased thickness of the interatrial septum, atrial walls and biatrial enlargement are common structural abnormalities identified by echocardiography in patients with CA [1]. In the early stage of the disease, the mitral inflow filling pattern obtained through pulsed-wave Doppler is characterised by a decreased early diastolic flow across the mitral valve (E wave) relative to the atrial wave (A wave), reflecting increased dependence on atrial contraction; with the disease progression, myocardial infiltration reduces LV wall compliance and elevates LA pressure, leading to worsening diastolic dysfunction [1].

In clinical settings, LA size is most reliably determined via 2D or, ideally, 3D echocardiography volume quantification. Conversely, assessing the anteroposterior LA diameter through M-mode or 2D parasternal long-axis views tends to result in a substantial underestimation of true LA dimensions [14]. Atrial dilatation reflects both primary atrial disease and secondary effects from elevated filling pressures, mitral regurgitation and AF. However, LA volumes’ evaluation presents some limitations: phasic LA volumes can be influenced by loading conditions, direct amyloid infiltration increases LA wall stiffness, limiting chamber enlargement, and LA dilatation has limited sensitivity for early disease. Consequently, deformation-based parameters offer incremental diagnostic and prognostic value in CA over LA volumes’ evaluation [3,4,14].

Impairments in LA function are typically manifested as reduced atrial contractility alongside an increase in LA stiffness [31]. Among currently available modalities, LA strain assessed by speckle-tracking echocardiography provides the most robust evidence for the evaluation of atrial involvement in CA, as it directly quantifies intrinsic LA myocardial deformation with relative independence from loading conditions and geometric assumptions, and with high feasibility and reproducibility [32]. Research involving CA patients has shown a gradual impairment across all three phases of atrial activity, specifically affecting the LA contraction, conduit, and reservoir functions [4,7]. Patients with wild-type ATTR-CA appear to exhibit more severe LA functional impairment compared with other CA etiologies, suggesting a subtype-specific impact of amyloid on atrial pathophysiology [7]. Interestingly, the LA reservoir strain demonstrated, in small cohorts, a role in differentiating CA from phenocopy conditions [33,34,35] and could potentially facilitate the early identification of sub-clinical disease among individuals carrying TTR mutations [36].

Several groups have investigated the role of atrial deformation analysis in predicting clinical outcomes. In a cohort of 149 ATTR-CA patients, Ozbay et al. showed that LA strain and LA stiffness demonstrated a weak correlation with LV diastolic dysfunction, suggesting a role of the primary infiltrative component of A-CM in CA [5]. Moreover, the authors observed that LA function parameters maintained an independent association with future thrombotic complications, even after adjusting for baseline AF presence or CHA_2_DS_2_-VASc scores [5]. In a population of 448 patients (50.2% AL-CA; 49.8% ATTR-CA) and no history of AF at diagnosis, Akintoye et al. showed that LA reservoir and contractile strain significantly predicted incident thrombotic event at median follow-up of 3.8 years [37]. Moreover, LA function parameters significantly improved current prognostic staging systems [37]. It is proposed by the authors that LA strain analysis should be integrated into the routine clinical management of individuals with AL or ATTR amyloid cardiomyopathy. Specifically, they advocate for considering preventive anticoagulation therapy when LA reservoir strain falls below 6.4% or contractile strain is less than 2.4%, regardless of whether AF is present [37].

Atrial functional assessment allows the identification of a subset of patients with “atrial electromechanical dissociation” (AEMD), defined as the absence of effective atrial mechanical contraction on speckle-tracking echocardiography despite the presence of sinus rhythm on surface ECG. In a cohort of 906 patients with ATTR-CA, Bandera et al. reported AEMD in 22.1% of patients in sinus rhythm [4]. Notably, AEMD conferred a similar prognosis to patients in AF in this population and warrants consideration of prophylactic anticoagulation [4]. According to research by Porcari et al., in a large cohort of 2310 patients with ATTR-CA individuals presenting with AEMD (identified in 13.2% of cases) or those in sinus rhythm with profound LA dysfunction (LA contractile strain <4%) exhibit cerebrovascular event rates that significantly exceed the standard thresholds for anticoagulation [38]. These high-risk phenotypes are identified as candidates who should be prioritized in future clinical trials evaluating prophylactic anticoagulation, according to the authors. These findings strongly support the concept that atrial mechanical dysfunction may have important prognostic implications in ATTR amyloidosis; however, they are hypothesis-generating and based on observational data. Accordingly, the potential role of prophylactic anticoagulation in patients with documented atrial mechanical dysfunction remains to be established and warrants prospective validation before clinical application can be recommended. In addition, further studies are needed to integrate imaging-derived parameters, including LA strain, into existing prognostic staging systems and to externally validate their incremental value. Nevertheless, these observations provide a rationale and conceptual framework for future trials of “mechanics-guided” anticoagulation, which may ultimately support a paradigm in which A-CM is directly targeted as a therapeutic substrate.

Of note, conduit and reservoir strain can be assessed irrespective of rhythm, whereas contractile strain requires organised atrial activity, limiting its use in patients with AF. Furthermore, technical constraints can sometimes hinder the feasibility of LA strain assessment. These include factors such as significant dropout of the atrial wall, LA foreshortening, suboptimal acoustic windows, or excessive cardiac displacement due to respiratory motion [4]. Recently, novel parameters such as LA stiffness [39] and atrial mechanical dispersion [40] have been introduced in CA, although their clinical role requires validation in larger cohorts.

#### 4.3.2. TEE

**Figure 3:**
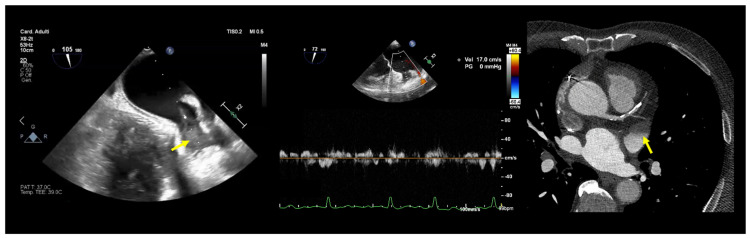
TEE in a patient with transthyretin cardiac amyloidosis: presence of left atrial appendage thrombus (arrow) and severe reduction in left atrial appendage pulsed-wave Doppler emptying velocity. Cardiac CT of the same patient, performed during follow-up, con-firming the presence of a stratified left atrial appendage thrombus (arrow).

In cardiac amyloidosis, the primary indications for TEE imaging include ruling out thrombi (Figure 3) and identifying potential cardioembolic sources [14]. Comprehensive interrogation in multiple imaging planes, supplemented by 3D acquisitions when available, is essential to confidently confirm or rule out thrombus and evaluate the presence of spontaneous echocardiographic contrast, characterised by dynamic, smoke-like swirling echoes within the LA and/or LAA, reflecting blood stasis and increased thrombogenicity. Moreover, impaired LAA mechanical function, commonly evaluated by pulsed-wave Doppler emptying velocities ≤ 20 cm/s (Figure 3), represents a well-established marker of thromboembolic risk [41].

Atrial structural and functional remodelling driven by amyloid infiltration promotes stasis, thrombus formation, and embolic events [42]. Irrespective of therapeutic anticoagulation status or arrhythmia duration, contemporary consensus suggests that TEE should be routinely conducted before cardioversion in cases of CA-associated AF [43,44]. This cautious strategy is supported by observational studies demonstrating a strikingly high prevalence of LAA thrombi in CA, leading to cancellation of up to 28% of scheduled cardioversions [45,46]. Nonetheless, emerging data suggested that in adequately anticoagulated CA patients, direct current cardioversion may be performed safely without pre-procedural TEE [47,48], challenging current practice recommendations. However, until larger, prospective cohorts better quantify residual thrombotic risk under optimal anticoagulation in this subset of patients, a conservative imaging-guided approach remains prudent [49]. Therefore, at present, a cautious strategy favouring routine pre-procedural TEE even in adequately anticoagulated CA patients appears appropriate, while recognising that future prospective data may modify this recommendation.

#### 4.3.3. CMR

As outlined above, CMR provides a comprehensive 3D assessment of LA anatomy and function and can identify atrial fibrosis using LGE imaging [14].

CMR steady-state free-precession (SSFP) cine sequences yield high blood–myocardium and epicardial–fat contrast, facilitating accurate definition of atrial endocardial contours and reliable quantification of LA dimensions and volumes [14]. Real-time cine sequence can be used if severe arrhythmia, such as AF, is present, overcoming the problem of ECG synchronisation. When assessing LA size and function, it is important to consider two different approaches: biplane (2D) versus volumetric (3D) approaches. The biplane area–length method uses 2- and 4-chamber cine views and enables straightforward quantification of LA volume and LAEF using a mathematical approximation; this approach is widely available and time-efficient. In contrast, 3D volumetric assessment requires a stack of short-axis or transverse slices, necessitating additional image acquisition and more intensive post-processing. Nevertheless, accumulating evidence suggests that 2D-based methods systematically underestimate LA volumes and may provide less accurate assessment of LAEF compared with 3D volumetric techniques, particularly in the presence of asymmetric atrial remodelling. To quantify LAEF, the difference between the largest and smallest LA volumes is expressed as a proportion of the maximum volume. Using this methodology, Aquaro et al. defined a LAEF ≤14% as a marker for advanced atrial dysfunction [50]. Through CMR assessments conducted on 80 consecutive CA patients—including 38 with AL and 42 with ATTR—it was found that three-quarters of the cohort exhibited severe LA contractile impairment. This condition was prevalent across both CA etiologies, regardless of AF status. Notably, such profound LA dysfunction was independently linked to a four-fold increase in the 3-year risk of cardiac mortality; furthermore, patients in sinus rhythm with an LAEF ≤14% exhibited even poorer clinical outcomes than those with documented AF [50]. Among patients with systemic AL amyloidosis, reduced LA emptying fraction was associated with additional markers of cardiac involvement and was predictive of poorer 2-year survival [51].

**Figure 4:**
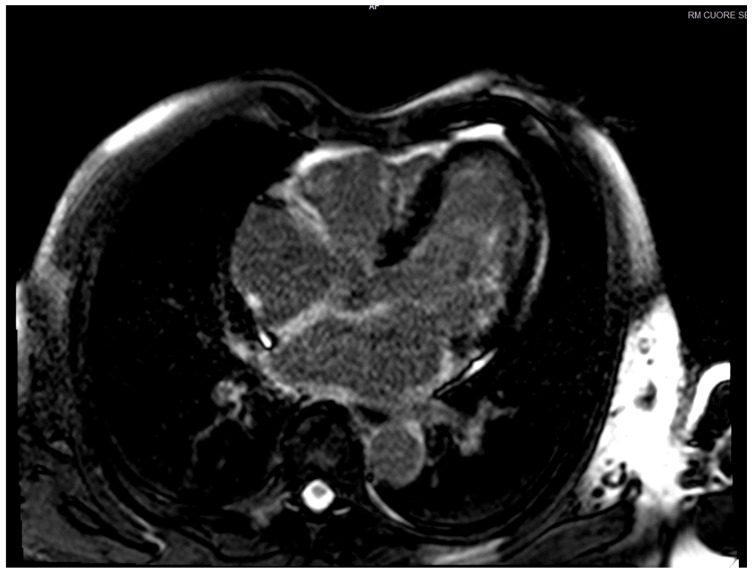
Diffuse late gadolinium enhancement (LGE) involving the left atrial wall in a patient with transthyretin cardiac amyloidosis.

CMR enables detailed tissue characterisation and detection of atrial fibrosis using high-resolution 3D-LGE imaging [52]. However, LGE assessment of the LA remains technically challenging [53]. Compared with the LV, the LA exhibits a more complex geometry, markedly thinner walls, and is enveloped by epicardial adipose tissue, making the use of conventional 2D-LGE with standard slice thickness suboptimal. Additionally, imaging the LA during its quiescent phase is critical to minimise motion-related artefacts. To overcome these limitations, 3D-LGE sequences with higher spatial resolution and respiratory motion compensation are needed. Currently, assessment of atrial LGE is primarily visual, typically limited to experienced centres, lacks standardised quantification, and is not feasible when image quality is suboptimal; moreover, substantial interobserver variability remains a key limitation, underscoring the need for further standardisation to support broader clinical implementation [52]. The presence of LA fibrosis in the atrium has been reported in various conditions such as AF, mitral valve disease and CA. In AF, some studies showed an association between LGE burden and AF recurrence following ablation [54,55]. Extensive atrial LGE due to amyloid deposition or fibrosis has been demonstrated in CA (Figure 4), which is associated with a marked reduction in LA emptying function [56]. In a study by Ozbay et al., compared to patients with other phenotypes of LV hypertrophy, patients with ATTR-CM showed more intense atrial LGE, with a more restrictive physiology reflected by less pronounced LA dilation despite more severe functional impairment and increased LA stiffness [5].

CMR has become an established imaging technique for the detection and characterization of LA and LAA thrombi, particularly through the use of delayed-enhancement CMR with long inversion time (TI) sequences [57]. According to a meta-analysis by Chen et al., delayed-enhancement CMR demonstrated the highest sensitivity, specificity, and overall diagnostic accuracy among the sequences studied [58].

The use of CMR feature tracking (CMR-FT) has gained prominence as a robust technique for evaluating LA strain, leveraging FT algorithms that utilize standard long-axis cine sequences [59]. By providing a non-invasive diagnostic tool, LA strain may facilitate the differentiation of CA from alternative hypertrophic phenotypes, as CA patients typically present with lower LA strain values than those with hypertrophic cardiomyopathy [60]. Evidence suggests that the LA reservoir strain derived from CMR-FT could potentially provide additive predictive value for all-cause mortality in patients with AL-associated CA, independent of other traditional markers [61]. Recently, a meta-analysis by García Méndez et al. (20 studies, 3260 patients) evaluated the prognostic value of LA function parameters assessed by TTE or CMR in patients with CA [62]. The analysis showed that in patients with CA, atrial strain measurements were significantly associated with all-cause mortality and the occurrence of AF. Nevertheless, since the quality of evidence regarding clinical outcomes remains graded as low to very low, routine implementation for prognostic assessment is not currently recommended. Future large-scale, multicentre prospective investigations are essential to formulate and validate precise risk prediction models that combine atrial parameters with clinical data and biomarkers, ultimately determining their practical utility in clinical management and follow-up [62].

Four-dimensional (4D) flow CMR enables detailed quantification of LA blood flow, offering potential novel biomarkers for thrombus formation and embolic stroke risk [63]. However, to the best of our knowledge, this technique has not been specifically investigated in populations of patients with CA.

Emerging approaches, supported by early feasibility studies, include LA T1 mapping to quantify extracellular volume (ECV) as a marker of diffuse atrial fibrosis, as well as black-blood CMR techniques to enhance visualisation and characterisation of LA wall thickness [64,65]. However, studies specifically directed to CA populations are lacking.

#### 4.3.4. Other Imaging Modalities (CCT, SPECT, PET)

CCT has historically had a limited role in CA, with no established utility for diagnosis or disease monitoring to date [66]. In pre-ablation assessment, for pulmonary vein anatomy imaging, CCT has higher spatial resolution than CMR. CCT represents a reliable alternative to TEE for thrombus detection (Figure 3); however, it necessitates an additional delayed scan after contrast, which may result in increased patient radiation exposure [67,68]. To date, no studies have characterized atrial involvement or associated outcomes in CA using CCT, despite the demonstrated utility of this modality in other A-CM settings for predicting both ablative success and stroke risk [69].

While bone-seeking radiotracer scintigraphy is now the primary tool for the non-invasive identification of ATTR-CA, detailed accounts of atrial characteristics remain scarce [69]. Although atrial radiotracer retention has been linked to confirmed histological CA and increased AF prevalence, a consensus on the formal grading or systematic evaluation of atrial uptake has yet to be established [70].

The diagnostic potential of PET in CA is currently being explored through novel tracers like 18F-florbetapir and 124I-evuzamitide. Preliminary data from early-phase studies indicate that these markers provide high-sensitivity uptake patterns and quantifiable metrics across a broad spectrum of amyloidosis, including ATTR, AL, and rarer subtypes [71]. To date, no studies have specifically investigated the use of these markers in A-CM.

### 4.4. Management of AF in CA

#### 4.4.1. Anticoagulation

AF in patients with CA carries an exceptionally high risk of stroke and systemic embolism. Multiple studies have reported a lack of significant correlation between the CHA_2_DS_2_-VASc score and LAA thrombus, underscoring its limited utility in predicting thromboembolic risk in ATTR-CM [72,73]. According to the latest ESC-HFA consensus statement, anticoagulation is always advised in patients with ATTR-CM and AF, regardless of their CHA2DS2-VASc score [43]. Intracardiac thrombus rates in AL-CA appear comparable, prompting current recommendations that all patients with AF and CA should be considered for oral anticoagulation irrespective of their score [74,75].

Despite maintaining sinus rhythm, individuals with CA face a persistent threat of cerebrovascular complications; however, a significant number of these cases go undiagnosed in clinical practice [76]. To date, clear guidance on initiating anticoagulation in patients with ATTR-CA who are in sinus rhythm is lacking, and anticoagulation may be advised according to the latest ESC-HFA consensus statement in individuals with evidence of LA dysfunction (reduced transmitral A wave <20 cm/s in the absence of a clear LV restrictive filling pattern, atrial standstill, or marked LA enlargement measuring >50 mm in diameter) [43]. In all cases, the risk–benefit profile should be assessed individually, particularly in frail patients with wild-type ATTR. Although observational data indicate that evaluating LA strain could improve risk assessment and identify ATTR-CM patients in sinus rhythm who might require advanced rhythm surveillance or preventative anticoagulation, these findings lack prospective validation. Future controlled trials are essential to confirm if LA strain-guided anticoagulation protocols actually lead to improved clinical outcomes [4,38].

Though large-scale randomized controlled trials are notably absent, existing observational and indirect data indicate that vitamin K antagonists (VKA) and direct oral anticoagulants (DOAC) have comparable efficacy in preventing thromboembolic events in ATTR-CA [43]. A systematic review and meta-analysis by Nong et al. reported that available evidence supports DOACs as a non-inferior alternative to VKAs for thromboembolism prevention in patients with AF and CA [77]. Current findings are constrained by the small cohorts and retrospective designs of existing research. Consequently, large-scale prospective investigations are needed to confirm these results and to develop robust clinical protocols that address the specific complexities of anticoagulation therapy in this vulnerable group [77]. Moreover, AL-CA poses unique challenges in anticoagulation regimen selection, due to the potential drug interactions (i.e., DOAC and dexamethasone) and the higher prothrombotic and bleeding risks associated with this condition [77]. Therefore, the choice of anticoagulant therapy should be individualised, taking into account the specific type of amyloidosis as well as concomitant treatments, given the heterogeneity in thromboembolic risk, bleeding propensity, and potential drug–drug interactions across different clinical settings.

Although data are limited, LAA closure may be considered in patients with ATTR-CA who have prohibitive bleeding risk or contraindications to anticoagulation [78].

#### 4.4.2. Rate and Rhythm Control

The choice between rate and rhythm control in CA remains a subject of significant debate, as current evidence is both sparse and conflicting. To date, no definitive survival advantage has been established for either management strategy. This lack of consensus is further complicated by the nature of the existing literature, which consists primarily of single-center, retrospective analyses. Therefore, according to the latest ESC-HFA consensus statement, the choice between a rate or rhythm control strategy should be individualised in ATTR-CA [43]. According to a recent French Nationwide Delphi Study, management strategies for AF and atrial flutter showed consistency across amyloidosis subtypes (ATTR and AL), and rhythm control strategy is favoured by the panel in early stages of the disease [79]. Indeed, AF has been associated with higher prevalence and incidence of heart failure in CA, as the loss of atrial contribution worsens functional status and elicits symptoms in a restrictive amyloidotic heart [24].

The drugs used for rate control in AF (beta-blockers, non-dihydropyridine calcium channel blockers, and digoxin) are usually poorly tolerated, as they may lessen the compensatory rate response to maintain adequate cardiac output in the amyloidotic heart [43]. A general agreement exists regarding the avoidance of non-dihydropyridine calcium channel blockers in this context; conversely, digoxin and beta-blockers may be considered for rate management, provided that bradycardia is strictly avoided [43]. The general advice is to start at low doses, with strict clinical monitoring of any signs of intolerance (hypotension, low cardiac output, clinical decompensation and/or conduction disorders); in case of digoxin use, it is mandatory to check serum levels, as toxicity could also occur at therapeutic serum levels [43].

Data on the success of rhythm control strategies are scarce [74]. Many experts favour the use of antiarrhythmic drugs (AADs), given the risks of drugs used for rate control, and amiodarone may be the antiarrhythmic of choice [43,80]. Due to the negative inotropic and chronotropic effects of drugs used to obtain rate and/or rhythm control, direct current cardioversion could be an option for the initial achievement of sinus rhythm and in symptomatic/unstable patients [74]. Given that intracardiac thrombosis is frequently observed in CA patients even when they are effectively anticoagulated, TEE is recommended prior to any cardioversion attempt. This recommendation overrides traditional clinical markers, such as an AF duration or adequate anticoagulation [42]. Because the incidence of complications in CA is notably higher than in standard populations, direct current cardioversion is considered a particularly high-risk strategy for patients with ATTR-CM [46]. The frequency of initial efficacy of direct current cardioversion appears to be similar between these patients and control groups, but long-term recurrences may be higher in those with cardiac amyloidosis [25,46,74]. The evidence base for the safety and effectiveness of pulmonary vein ablation in CA is currently sparse, consisting primarily of small, single-center studies with a high incidence of AF recurrence [43]. Consequently, clinical consensus states that catheter ablation should be evaluated on an individualized, case-by-case basis [43,74].

If pharmacological therapy is ineffective, atrio-ventricular node ablation with permanent pacemaker implantation could be a safe option; however, patient selection must be strict. In order to mitigate the risk of decompensation, the pacing rate should be set relatively high, typically 80–90 bpm; furthermore, cardiac resynchronisation therapy is generally favored over standard RV pacing in these clinical scenarios [43,74].

#### 4.4.3. Role of Disease-Modifying Drugs and Other Drugs in AF Risk

In a retrospective review of 473 patients with ATTR-CA by Girvin et al., tafamidis was associated with a 57% reduction in the risk of atrial fibrillation development, after adjustment for the measured confounders (including age, sex, LV ejection fraction, obesity, hypertension, and LA diameter) [81]. Therefore, this study suggests that in patients diagnosed with ATTR-CA, the administration of tafamidis may lower the occurrence of AF: directly, reducing the deposition of amyloid via tetramer stabilisation and, indirectly, slowing the progression to diastolic heart failure and LA dilation. Furthermore, the positive effects of the drug on the autonomic nervous system may alleviate the risk of AF [82].

Conversely, an analysis of a real-world cohort of 235 ATTR-CA patients initially free of AF revealed that tafamidis did not lower the risk of incident AF, despite providing a clear survival benefit [83]. These conflicting results likely reflect differences in study design, patient populations, and residual confounding, and underscore the need for further prospective studies to clarify whether tafamidis has a true impact on AF incidence in ATTR-CA.

## 5. Anderson-Fabry Disease

### 5.1. Prevalence, Pathogenesis and Clinical Impact of AF in FD

FD is an X-linked inherited lysosomal storage disorder caused by pathogenic variants in the galactosidase-α gene (GLA), resulting in a total or partial deficiency of the enzyme α-galactosidase A. The subsequent accumulation of globotriaosylceramide (Gb3) and globotriaosylsphingosine (lyso-Gb3) causes multi-organ dysfunction through both direct lysosomal stress and secondary signaling pathways [84]. Clinically, this manifests as life-threatening complications involving the heart, kidneys, and brain, significantly reducing patient life expectancy and quality of life [84]. Within the myocardium, the deposition of glycosphingolipids infiltrates every cardiac cell lineage. This cellular infiltration triggers LV hypertrophy and promotes inflammatory pathways and myocardial fibrosis that predispose the heart to arrhythmias [85,86].

AF is the most frequently reported supraventricular arrhythmia in FD, yet the cellular mechanisms accounting for this are unknown. The reported prevalence ranges between 3 and 21%, and the incidence is around 6% per year; persistent AF is present in 3.9% of the patients and paroxysmal form in 13.3% of the patients [87,88]. In a small cohort of 68 FD patients with a mean follow-up of 1.9 years, age emerged as the only independent predictor of AF or paroxysmal AF [89]. The development of AF may precipitate acute hemodynamic decline and significantly elevate the likelihood of cerebrovascular accidents [90].

Multiple structural abnormalities are implicated in the pathogenesis of AF in this disease. In this context, the development of A-CM has been proposed in Fabry disease, likely reflecting two complementary mechanisms. First, histopathological studies have demonstrated sphingolipid deposition within the atria, promoting atrial remodelling that may precede LV hypertrophy and serves as an early and prognostically useful marker of cardiac involvement [9]. In addition, LV hypertrophy increases LV end-diastolic pressure, which is transmitted to the LA, leading to dilation and impaired LA function, thereby promoting atrial arrhythmogenesis [91]. Atrial and ventricular arrhythmias, along with distinctive ECG patterns in FD, may be attributed to the altered electrical properties of cardiomyocytes in FD. Specifically, in vitro data highlight that enhanced sodium and calcium channel currents lead to the development of higher and shorter spontaneous action potentials [86,92]. Overall, the pathogenesis of AF in FD appears to be multifactorial, likely involving the interplay of sphingolipid deposition-related atrial remodelling, increased ventricular filling pressures, and intrinsic electrophysiological alterations, although the relative contribution of these mechanisms remains to be fully elucidated.

### 5.2. LA Multimodality Imaging in FD

#### 5.2.1. TTE

TTE is essential to assess LV hypertrophy, abnormal LV strain and diastolic dysfunction [93]. This imaging technique also allows an early detection of the presence of atrial dilatation and dysfunction, which may be a trigger for AF. In this context, speckle-tracking echocardiography is a sensitive tool to ascertain early atrial dysfunction. Evidence from cross-sectional research has highlighted a reduction in LA function among individuals diagnosed with FD. Indeed, as shown by Pichette et al. in a small cohort of 50 patients with FD, all LA strain phases were affected in FD patients compared with 50 healthy control subjects; furthermore, over a 4-year follow-up, the risk of stroke and the occurrence of new onset AF were significantly linked to baseline measurements of early diastolic strain and peak atrial longitudinal strain, with atrial functional parameters surpassing conventional echocardiographic parameters and clinical characteristics [94]. In a cohort of 33 FD patients who underwent TTE before starting enzyme replacement therapy, the authors demonstrated LA enlargement and reduced LA compliance that occurs before the development of LV hypertrophy, suggesting a role for a primary LA cardiomyopathy in FD [9]. In a recent meta-analysis including fourteen studies and 1713 FD patients, multiple imaging metrics (among others, LA volume indexed) have been identified as significant predictors of negative clinical outcomes [95]. These findings reinforce the utility of multimodality cardiovascular imaging as a prognostic tool for individuals with FD; nevertheless, significant discrepancies remain in how endpoints are defined and imaging procedures are conducted. To address this, large-scale multicenter collaborations are essential to establish uniform data collection methods, which will facilitate the harmonization and validation of current and future imaging biomarkers [95].

Overall, while these observations are derived from relatively small and heterogeneous cohorts and should be interpreted with caution, data consistently point toward a potential role for LA size and functional assessment in the early detection of cardiac involvement in FD; however, this hypothesis requires validation in adequately powered, prospective, multicentre studies with standardised imaging protocols.

#### 5.2.2. CMR

CMR is considered an essential imaging modality for both the diagnosis and the staging of cardiac FD. Typical findings include ventricular LGE (initially involving the basal inferolateral wall), which may be associated with evidence of myocardial inflammation (seen through T2 mapping sequences) and reduced native T1 values (which likely reflect myocardial glycosphingolipid storage and may be present before the development of significant LV hypertrophy) [96]. FD is predominantly an intracellular storage disorder, in contrast to CA; accordingly, extracellular volume is usually normal, except in regions demonstrating LGE or in later stages of the disease [96].

In FD, CMR studies focusing on atrial involvement have predominantly concentrated on the assessment of LA function using CMR-FT techniques. In a CMR-study involving 58 patients with genetically proven FD compared to age and sex-matched healthy controls, Halfmann et al. showed that CMR-derived LA reservoir strain acts as a reliable marker for distinguishing early-phase FD from healthy controls, a distinction that atrial volumes and T1 mapping were unable to provide in this cohort [97]. Furthermore, atrial strain offered superior diagnostic utility for early FD compared to multiparametric models that include ventricular strain, and a significant correlation was found between LA strain and disease severity (quantified by myocardial T1 mapping and LV mass) [97]. These data suggest that assessing atrial deformation is paramount for evaluating patients with suspected cardiac involvement in FD [97]. Even before LV hypertrophy or diastolic dysfunction become apparent, CMR-FT assessment of LA function reveals subclinical atrial impairment in FD [98]. Bernardini et al. identified that this early dysfunction occurs prior to significant ventricular remodeling, suggesting that atrial metrics are highly sensitive in the initial stages of the disease [98]. These data are consistent with the concept of an LA disease directly caused by Gb3 deposition, primarily affecting atrial compliance. On the other hand, Cheepvasarach et al. identified that while LA strain remains within normal limits during the initial phases of FD, it becomes impaired once LV hypertrophy develops [99]. In their cohort of 214 patients, the decline in LA strain was correlated to markers of disease severity, including native T1, LV global longitudinal strain and LV mass. These findings suggest that atrial strain impairment in FD is a feature of abnormal LV mechanics rather than a direct effect of isolated atrial sphingolipid deposition.

Taken together, these apparently conflicting findings underscore the ongoing uncertainty regarding the primary mechanisms underlying LA dysfunction in FD. Further large-scale, multicentre prospective studies and dedicated registries, ideally incorporating standardised multimodality imaging protocols and longitudinal follow-up, are warranted to clarify the pathophysiological substrate and to better define the clinical and prognostic role of LA functional assessment in this setting.

### 5.3. Management of AF in FD

#### 5.3.1. Anticoagulation

None of the currently available stroke risk stratification scores have been validated in FD, and extrapolation from hypertrophic cardiomyopathy indicates that their use in this population is inappropriate [84]. According to the latest Expert Consensus Document on FD, all patients with FD and AF or atrial flutter should receive anticoagulation with DOAC or vitamin K antagonists (VKA), unless contraindicated [84]. In patients with appropriate renal clearance, DOAC therapy is favored over vitamin K antagonists for stroke prophylaxis; this preference is driven by the need to mitigate the incidence of warfarin-related nephropathy and minimize the likelihood of cerebral microbleeds [84,100,101]. In cases where anticoagulant administration is not feasible, clinicians may consider LA appendage occlusion as a secondary strategy to prevent thromboembolic events [84].

#### 5.3.2. Rhythm and Rate Control

Patients with FD poorly tolerate supraventricular arrhythmias due to impaired diastolic filling. According to the latest Expert Consensus Document on FD, maintenance of sinus rhythm rather than rate control is recommended for patients with FD and AF [84]. While pharmacological agents, ablation, or electrical cardioversion may be employed, the long-term efficacy of these strategies is frequently limited. This is primarily due to the progressive nature of the disease, which involves significant atrial dilatation, structural reorganization, and functional decline [85]. Long-term use of amiodarone is generally contraindicated in patients with FD [84]. Indeed, the drug may alter lysosomal pH and enzyme activity, and chronic therapy may induce phospholipidosis and potentially reduce the effect of enzyme replacement therapy (ERT), complicating the overall treatment strategy [84,102,103]. Sotalol is contraindicated in the presence of acute heart failure and severe renal impairment (creatinine clearance < 10 mL/min). Regarding flecainide, caution is needed if the estimated glomerular filtration rate is under 35 mL/min [84]. Although there is now general support for early ablation in all patients presenting with AF, there are still concerns regarding long-term efficacy in FD [85].

In patients in whom rate control is chosen, the additional concern regards the co-existing risk of bradyarrhythmia and conduction disease, common in FD. Beta blockers or calcium channel antagonists are generally favoured as the first line for rate-limiting strategies [85]. Periodic Holter monitoring is essential for assessing the efficacy of rate control, especially considering the inherent risk of bradyarrhythmias and impaired electrical conduction in these individuals [84,104].

#### 5.3.3. Role of Disease-Modifying Drugs in AF Risk

Biweekly intravenous administration of agalsidase beta or agalsidase alfa has been the standard for ERT since these recombinant human α-Gal A formulations entered clinical practice in the early 2000s [90]. The efficacy of ERT is largely dependent on the timing of intervention, with superior results observed when treatment begins early. Conversely, once the hallmark signs of FD cardiomyopathy manifest, accompanied by an elevated risk of arrhythmias, the capacity for ERT to modify the course of the disease is notably diminished [87]. Research by Pichette and colleagues demonstrated that ERT positively influenced LA function (evaluated by speckle-tracking echocardiography) after 12 months of treatment. These findings suggest that ERT may potentially arrest or even reverse the advancement of A-CM in this patient group [94]. However, there is currently no definitive evidence that ERT could reduce the burden and risk of AF in FD [105], and further studies are needed.

The reversible, active-site inhibitor deoxygalactonojirimycin (migalastat) is a pharmacological chaperone that increases in vitro and in vivo activity by accelerating the transport and maturation of the residual.

α-Gal A; it can be administered in FD patients with certain GLA gene missense mutations called “amenable” [106]. The therapeutic impact of migalastat appears constrained once cardiomyopathy is established, mirroring the trends seen with ERT. Notably, there remains a lack of evidence to suggest that this chaperone therapy provides any benefit in reducing AF burden and risk [107].

## 6. Cardiac Sarcoidosis

### 6.1. Prevalence, Pathogenesis and Clinical Impact of AF in CS

CS is an inflammatory disease arising from dysregulated cellular immunity leading to non-caseating granuloma formation within myocardial tissue, which, as the disease progresses, is gradually replaced by interstitial collagen deposition and patchy myocardial fibrosis [108]. Clinically overt cardiac sarcoidosis is rare, as it is identified in roughly 5–7% of patients with systemic sarcoidosis; nevertheless, cardiac involvement may represent the first, and sometimes the sole, clinical manifestation of the disease [109].

AF is common among patients with CS, with contemporary meta-analyses reporting a prevalence of around 20% and an incidence exceeding 10% at medium-term follow-up [110,111]. According to the latest Clinical Consensus Statement on CS by Sharma et al., the mechanisms underlying atrial arrhythmia in patients with CS are multifactorial [112]. LV involvement leads to increased LA pressure and dilatation, promoting AF as in other forms of heart failure [113,114]. Pulmonary hypertension related to sarcoid-lung disease can also cause right atrial enlargement, predisposing to typical atrial flutter [115]. In addition, direct atrial granulomatous infiltration may cause inflammation followed by fibrosis and scarring, which disrupts atrial conduction and creates a substrate for re-entrant atrial arrhythmias, including both AF and atrial flutter [108,112,116,117,118,119]. Since both increased atrial FDG uptake on PET and the presence of LGE on cardiac MRI are associated with elevated arrhythmia risk in CS, these findings underscore the importance of this inflammatory–fibrotic substrate in the disease’s pathophysiology [110,112,120,121,122]. Furthermore, traditional risk factors such as advancing age, diabetes mellitus, and hypertension likely contribute to a heightened susceptibility to atrial arrhythmias [109,122]. Clinically, AF denotes a high-risk CS phenotype, as it was associated with a higher incidence of heart-failure hospitalisations and reduced survival during the follow-up [111,123]. Moreover, as expected, CS patients with AF show increased risk of stroke events [110]. Thus, AF not only reflects atrial involvement but also contributes independently to adverse outcomes in this disease.

### 6.2. LA Multimodality Imaging in CS

Current evidence on multimodality atrial imaging in CS is largely derived from small, single-centre observational studies and retrospective cohorts, reflecting the relative rarity of the disease and the consequent difficulty in conducting large, prospective, controlled investigations.

TTE provides the most accessible tool for evaluating LA involvement in CS. In a cohort of 172 patients with sarcoidosis, Cameli et al. showed that global peak LA longitudinal strain was significantly lower at baseline in patients experiencing a relapse of the disease during follow-up [124]. Due to the limitations inherent in a single-institution, retrospective analysis, these intriguing results require cautious interpretation. Although the parameter appears promising for the prognostic assessment of sarcoidosis, more robust evidence is needed to confirm its clinical utility. As the disease progresses, atrial fibrosis, together with diastolic dysfunction and increased filling pressures secondary to LV involvement, leads to LA dilatation and worsened mechanics [125,126].

Data regarding atrial fibrosis, dilatation and functional assessment by CMR and 18F-FDG PET in CS remain limited to case reports or small observational studies [110,120,121,122]. Habibi and colleagues demonstrated in a study of fifty CS patients that AF was independently linked to reductions in both active and total LA ejection fraction evaluated through CMR-FT [121]. Their findings also suggested a possible connection between atrial FDG uptake and AF, despite the fact that most AF patients presented with multiple risk factors but lacked signs of active inflammation [121]. While the small sample size and retrospective approach represent significant study limitations, the results collectively suggest that CMR and PET imaging may provide valuable contributions to identifying CS patients at risk of future AF. Yodogawa and colleagues, in an analysis of 62 individuals with CS, demonstrated that atrial FDG accumulation was a frequent finding in this population. Their data further revealed a robust correlation between atrial uptake and the incidence of atrial arrhythmias [122]. The role of atrial inflammation as a primary predictor of arrhythmogenesis in CS was highlighted by Niemelä et al. following an analysis of 118 patients [110]. Starting from the time of the initial PET study, patients with metabolic evidence of atrial involvement faced a substantially higher risk of future AF compared to those without uptake (55% vs. 18% at 5 years). These findings underscore that metabolic activity serves as a more robust independent risk factor than traditional structural markers, such as CMR-derived LA enlargement or clinical comorbidities (like sleep apnea) [110].

While these findings underscore the significant potential of CMR and 18F-FDG PET in identifying atrial involvement in CS, current evidence remains largely exploratory and constrained by limited cohort sizes; consequently, large-scale multicentre prospective studies are warranted to establish robust clinical paradigms. Ultimately, refining atrial scar quantification through advanced CMR sequences and utilising longitudinal PET imaging to evaluate therapeutic response may provide the necessary precision to optimise AF risk stratification and management in patients with CS.

### 6.3. Management of AF in CS

Current general advice for rate/rhythm control and thromboembolism prevention in AF patients also applies to CS [109,127]. The data on the antiarrhythmic medication in CS is scarce. As highlighted in a recent clinical consensus statement, class I antiarrhythmic agents should be avoided in cardiac sarcoidosis [112]. Class III agents are generally used; however, sotalol and dronedarone are not recommended in the presence of reduced left ventricular ejection fraction [112]. Furthermore, while amiodarone may be considered an appropriate option in isolated cardiac sarcoidosis, its use might be deferred in patients with advanced pulmonary sarcoidosis, given the risk of pulmonary toxicity associated with this drug [128].

Catheter ablation represents a feasible rhythm-control strategy, although available evidence is derived from small observational series. Pulmonary vein isolation is safe and associated with symptom improvement and an arrhythmia-free survival comparable to non-inflammatory controls [129,130]. However, atrial arrhythmias in CS frequently arise from a complex atrial substrate, with patchy atrial low-voltage areas and non-pulmonary vein triggers [130]. While extended ablation beyond pulmonary veins should be individualised based on electroanatomical mapping, routine extensive ablation is not currently supported by evidence [109]. While more robust longitudinal research is required to fully define the role of catheter ablation in CS-related AF, the inherent challenges of pharmacological rhythm control in these patients make an invasive approach a justifiable alternative. Consequently, ablation should be considered for symptomatic individuals in whom maintaining sinus rhythm is a primary clinical objective [112].

Although data are sparse, individual case reports suggest that immunosuppression can effectively lower the frequency of AF episodes in CS patients [131,132]. It may be speculated that the decrease in myocardial inflammation may attenuate atrial arrhythmogenesis [133], but this hypothesis requires confirmation in larger, systematic studies. Thus, the routine use of immunosuppression for AF as the sole manifestation of CS is currently not recommended [109].

## 7. Iron Overload Cardiomyopathy

### 7.1. Prevalence, Pathogenesis and Clinical Impact of AF in IOC

IOC arises from systemic iron accumulation secondary to hereditary increased gastrointestinal absorption (hemochromatosis) or frequent administration of red blood cell transfusions (hemosiderosis). AF, mainly paroxysmal, represents a common arrhythmic manifestation, with reported prevalence ranging from 2% to 30% across cohorts, largely influenced by phenotype and disease severity [134,135]. AF is uncommon in asymptomatic young patients with hereditary disease but becomes increasingly prevalent with older age and progressive myocardial siderosis [136,137]. Consequently, AF occurs with greater frequency in advanced cases of IOC and is linked to a more severe symptom profile; furthermore, its presence is associated with poorer in-hospital prognoses, specifically characterized by extended durations of stay and increased mortality rates [135]. Excess intracellular iron catalyses the production of highly reactive hydroxyl radicals, ultimately causing cardiomyocyte injury and interstitial fibrosis [138,139]; this process, together with the direct pro-arrhythmic effect of iron through impaired calcium-channel function and conduction disturbances, leads to a primary A-CM and predisposes to AF [140]. Of note, iron accumulation in the ventricles precedes the atrial myocardium [141]; thus, increased LV filling pressures and diastolic dysfunction contribute to atrial remodelling and AF [139]. Extra-cardiac involvement also promotes AF, including inflammation, metabolic comorbidities such as diabetes and autonomous nervous system dysfunction [134]. Consequently, increased myocardial iron burden on CMR is a strong predictor of arrhythmic events, outperforming serum ferritin and liver iron; other predictors include LV diastolic dysfunction and LA enlargement and dysfunction, together with known AF risk factors like older age and diabetes [137,138].

### 7.2. LA Multimodality Imaging in IOC

Current evidence on multimodality atrial imaging in IOC is largely derived from small, single-centre, observational studies. Regarding TTE, in a report by Kostopoulou et al., an increase in the E/e’ ratio occurred earlier in IOC patients with transfusion-dependent iron overload and preserved LV ejection fraction compared with controls [142]. Other indices of diastolic dysfunction may also be impaired, including peak systolic and peak diastolic early filling tissue Doppler waves [143]. TTE allows the evaluation of atrial functional parameters through speckle-tracking analysis. Saad and colleagues demonstrated that alterations in LA deformation may manifest before the onset of overt cardiomyopathy in individuals with hereditary hemochromatosis [144]. Their research identified that even asymptomatic patients in the early stages of the disease exhibited reduced LA reservoir and conduit strains, along with a decreased LA conduit strain rate compared to healthy controls. Notably, the LA stiffness index, defined as the ratio of E/e′ to LA reservoir strain, was markedly elevated in the hemochromatosis cohort, even when LA dimensions and emptying fractions remained within normal limits. In an observational study on patients with transfusion-dependent β-thalassemia, patients with a history of AF showed significantly lower values of global longitudinal strain and peak LA longitudinal strain compared to those without AF, and the peak atrial longitudinal strain showed high discriminative ability with an optimal cut-off value of 25.9% to detect those with a history of AF [145]. The use of speckle-tracking echocardiography shows great promise for the subclinical detection of atrial dysfunction and the prediction of arrhythmic risk within the IOC population. Nevertheless, while current data are encouraging, the clinical utility of this technique must be further substantiated by expansive, prospective trials to confirm its reliability as a diagnostic standard.

CMR has a paramount role in assessing IOC, as the T2* technique allows non-invasive quantification of iron overload and can guide iron chelation therapy. Assessing atrial iron deposition via T2*-CMR remains a significant challenge due to the thinness of the atrial myocardium, which often leads to inaccurate readings from partial volume effects [137]. Therefore, despite the clinical importance of myocardial T2* in predicting heart failure and arrhythmias, to the best of our knowledge, specific data on atrial T2* values are currently unavailable. Consequently, while the atria may be more vulnerable to iron-induced arrhythmias than the ventricles, current non-invasive MR techniques lack the robustness required to accurately quantify atrial iron loading [137].

In a study including 264 patients with β-thalassemia major, CMR-FT-derived LA strain parameters were significantly reduced relative to healthy subjects and demonstrated a strong association with adverse cardiac outcomes, particularly heart failure and arrhythmic events, exceeding the predictive value of myocardial iron quantification (evaluated using myocardial LV-T2* values) [146]. No relationship was observed between LA strain parameters and myocardial iron overload, possibly because iron accumulation was generally modest in this cohort, likely reflecting effective transfusion management and chelation treatment. These findings suggest that the LA strain could function as an additional CMR marker, which may potentially improve risk stratification in this population [146]. However, the integration of these metrics into standard clinical practice requires validation through larger, longitudinal studies to confirm their long-term predictive accuracy. Overall, CMR plays a central role in the evaluation of IOC; however, atrial-specific imaging markers remain limited and technically challenging, highlighting the need for further dedicated studies to better characterise atrial involvement and its clinical implications.

### 7.3. Management of AF in IOC

The management of AF in IOC patients follows the general indications as for other types of dilated or restrictive cardiomyopathies, as disease-specific randomised evidence is lacking [75]. Of note, AF patients with restrictive cardiomyopathy should receive anticoagulation, regardless of their thromboembolic risk; this applies particularly to those with β-thalassemia, due to the chronic hypercoagulability state [134]. Furthermore, adequate treatment of comorbidities secondary to systemic iron overload, such as diabetes, may reduce AF burden [127].

A central aspect of AF management in IOC is aetiology-directed therapy. Significant reductions in cardiac events and arrhythmias are achievable through early myocardial iron clearance via chelation or phlebotomy. However, the clinical success of these interventions is heavily dependent on patient adherence; indeed, low compliance with chelation protocols is one of the most reliable predictors of arrhythmic risk in this population [137,147].

The IOC-AF population tends to be younger than its non-IOC counterpart; therefore, rhythm control should be favoured to improve outcomes [127]. Class I AADs are generally discouraged in patients with significant cardiomyopathies, while amiodarone is burdened by significant side effects, which may add to coexisting iron-related organ damage (e.g., thyroid, liver) [140]. Catheter ablation may be considered in selected patients with symptomatic AF refractory to medical therapy, although A-CM may limit its efficacy; moreover, procedural strategies should be tailored to the degree of atrial remodelling, as no data support routine extensive substrate modification beyond pulmonary vein isolation [148].

Rate control with beta-blockers is commonly employed, although their tolerance may be reduced in restrictive physiology. Non-dihydropyridine calcium-channel blockers can be considered in patients with preserved ventricular function. Interestingly, beyond rate control, experimental data suggest that L-type calcium-channel blockade may reduce intracellular iron accumulation and oxidative injury; however, whether these effects translate into clinically meaningful modification of atrial remodelling in humans remains to be established [149].

## 8. Conclusions, Limitations and Future Directions

A-CM has emerged as a unifying pathophysiological substrate underlying a broad spectrum of adverse cardiovascular outcomes, including AF, stroke, and heart failure. Increasing evidence suggests that this paradigm may be particularly relevant in Inf-CM, a group of conditions characterized by a high burden of systolic and diastolic LV dysfunction and a markedly elevated thromboembolic risk. To date, multi-modality cardiac imaging in Inf-CM has largely focused on ventricular involvement, providing crucial insights into diagnosis, staging, and prognosis. However, growing data indicate that atrial chambers are deeply affected, both as a primary manifestation of the infiltrative process and as a secondary consequence of ventricular and hemodynamic abnormalities. Atrial involvement likely substantially contributes to the arrhythmic and thromboembolic complications observed in these patients, and to heart failure progression. Importantly, current diagnostic and therapeutic approaches remain largely driven by the specific infiltrative cardiomyopathy subtype, with limited incorporation of atrial structural and functional markers into clinical decision-making. The available evidence supporting the role of A-CM imaging markers in Inf-CM is predominantly derived from observational studies, often limited by small sample sizes and single-center designs. Accordingly, prospective studies and large registries are needed in order to determine the incremental prognostic value of atrial imaging parameters, ultimately enabling their integration into robust, clinically actionable risk scores. In parallel, advances in automated deformation analysis and machine learning algorithms hold promise for enhancing the reproducibility and clinical applicability of atrial assessment, facilitating its incorporation into routine workflows.

A more comprehensive approach integrating atrial and ventricular imaging may enable earlier identification of patients prone to developing AF and heart failure, facilitating more targeted preventive strategies. Eventually, the advent of disease-modifying therapies in several Inf-CM raises the critical question of whether these treatments can favorably impact A-CM itself, ultimately translating into a reduction in AF, heart failure, and thromboembolic events.

A shift toward an integrated, multi-chamber characterization of cardiomyopathy is essential to refine risk stratification, guide therapeutic decision-making, and ultimately enable a more precise and individualized approach to Inf-CM.

## Figures and Tables

**Table 1:** Main atrial-specific findings in Inf-CM, and strengths and limitations of each non-invasive imaging modality for atrial assessment in Inf-CM.

	TTE	TEE	CMR	CCT	Scintigraphy	PET
Specific LA Findings in Inf-CM	Atrial dilatation (quantitative evaluation) Reduced LA function (LAEF) Reduced LA strain (all phases) Increased LAP Increased inter-atrial septum and atrial wall thickness (in CA)	Atrial dilatation (qualitative evaluation) LAA disfunction Increased inter-atrial septum and atrial wall thickness (in CA)	Atrial dilatation (quantitative evaluation) Reduced LA function (LAEF) Reduced LA strain (all phases) Increased inter-atrial septum and atrial wall thickness (in CA) LA fibrosis (LGE)	Atrial dilatation (quantitative evaluation)	Visual evidence of increased atrial uptake (in CA)	Atrial FDG uptake (in CS)
LA volume and morphology	+++	+	+++	+++	-	-
LA function (phasic function/strain)	+++	-	+++	+	-	-
LAA morphology, function and thrombus exclusion	-	+++	++	+++ (no assessment of LAA function)	-	-
LA inflammation/fibrosis	-	-	++	-	-	++
Strengths	Available Low cost No radiation No contrast	Gold standard for thrombus/SEC detection Available No radiation No contrast	Comprehensive 3D evaluation of LA anatomy, structure, and function Assessment of LA fibrosis	Available Low relative cost	Bisphosphonate scintigraphy with central role in ATTR-CA diagnostic pathway	Central role of FDG-PET in CS
Limitations	Depends on acoustic window Limited in patients not in SR	Invasive No LA volume assessment	Limited in specific settings (CKD, claustrophobia) Cost and accessibility	No clear role in InfCM Radiation exposure Contrast (limitation in CKD patients)	Very limited role for atrial assessment	Limited role for atrial assessment

Inf-CM, Infiltrative Cardiomyopathies; LA, left atrium; LAA, left atrial appendage; LAEF, left atrial ejection fraction; CA, cardiac amyloidosis; LAP, left atrial pressure; SR, sinus rhythm; SEC, spontaneous echo contrast;. LGE, late gadolinium enhancement;CKD, chronic kidney disease, ATTR-CA, transthyretin amyloid cardiomyopathy; FDG-PET, fluorodeoxyglucose-positron emission tomography; CS, cardiac sarcoidosis; TTE, transthoracic echocardiography; TEE, transesophageal echocardiography; CMR, Cardiovascular magnetic resonance; CCT, Cardiac computed tomography. (-) not suitable/not recommended; (+) limited suitability; (++) moderately suitable; (+++) highly suitable/preferred modality.

## Data Availability

No new data were generated during this study.

## References

[1] Kottam A., Hanneman K., Schenone A., Daubert M.A., Sidhu G.D., Gropler R.J., Garcia M.J., on behalf of the American Heart Association Council on Cardiovascular Radiology and Intervention (2023). State-of-the-Art Imaging of Infiltrative Cardiomyopathies: A Scientific Statement from the American Heart Association. Circ Cardiovasc. Imaging.

[2] Vad O.B., van Vreeswijk N., Yassin A.S., Blaauw Y., Paludan-Müller C., Kanters J.K., Graff C., Schotten U., Benjamin E.J., Svendsen J.H. (2026). Atrial Cardiomyopathy: Markers and Outcomes. Eur. Heart J..

[3] Camilli M., D’Elia E., Hulot J.S., Iacoviello M., Sisti N., Tica O., Tokmakova M., Van den Eynde J., Weerts J., Zakeri R. (2026). Role of Imaging Techniques in Monitoring Atrial Cardiomyopathy and Atrial Failure: A Scientific Statement. ESC Heart Fail..

[4] Bandera F., Martone R., Chacko L., Ganesananthan S., Gilbertson J.A., Ponticos M., Lane T., Martinez-Naharro A., Whelan C., Quarta C. (2022). Clinical Importance of Left Atrial Infiltration in Cardiac Transthyretin Amyloidosis. JACC Cardiovasc. Imaging.

[5] Ozbay B., Rearick C., Satyavolu B.S., Soman P., Wong T.C., Starr M., Pillai B., Zhu J., Azhar A.Z., Katz W.E. (2025). Primary Left Atrial Cardiopathy in Transthyretin Amyloidosis Cardiomyopathy by Multimodality Imaging. JACC Cardiovasc. Imaging.

[6] Modesto K.M., Dispenzieri A., Cauduro S.A., Lacy M., Khandheria B.K., Pellikka P.A., Belohlavek M., Seward J.B., Kyle R., Tajik A.J. (2005). Left Atrial Myopathy in Cardiac Amyloidosis: Implications of Novel Echocardiographic Techniques. Eur. Heart J..

[7] Nochioka K., Quarta C.C., Claggett B., Roca G.Q., Rapezzi C., Falk R.H., Solomon S.D. (2017). Left Atrial Structure and Function in Cardiac Amyloidosis. Eur. Heart J.-Cardiovasc. Imaging.

[8] Sheppard M.N., Cane P., Florio R., Kavantzas N., Close L., Shah J., Lee P., Elliott P. (2010). A Detailed Pathologic Examination of Heart Tissue from Three Older Patients with Anderson-Fabry Disease on Enzyme Replacement Therapy. Cardiovasc. Pathol..

[9] Boyd A.C., Lo Q., Devine K., Tchan M.C., Sillence D.O., Sadick N., Richards D.A.B., Thomas L. (2013). Left Atrial Enlargement and Reduced Atrial Compliance Occurs Early in Fabry Cardiomyopathy. J. Am. Soc. Echocardiogr..

[10] Mehta D., Willner J.M., Akhrass P.R. (2015). Atrial Fibrillation in Cardiac Sarcoidosis. J. Atr. Fibrillation.

[11] Ahmed G., Rathi S., Sidhu H.K., Muzaffar M., Wajid M.H., Kumari K., Fakhor H., Attia N.M., Majumder K., Kumar V. (2023). Paroxysmal Atrial Fibrillation and Hemochromatosis: A Narrative Review. Ann. Med. Surg..

[12] Schubert M.R., Sinner M.F. (2026). Understanding Atrial Cardiomyopathy: A Missing Link in the Prevention of Atrial Fibrillation, Heart Failure, and Stroke?. Eur. Heart J..

[13] Weerts J., Țica O., Aranyo J., Basile C., Borizanova-Petkova A., Borovac J.A., Camilli M., Eichenlaub M., Fiori E., Van Loon T. (2025). Atrial Cardiomyopathy: From Healthy Atria to Atrial Failure. A Clinical Consensus Statement of the Heart Failure Association of the ESC. Eur. J. Heart Fail..

[14] Sade L.E., Faletra F.F., Pontone G., Gerber B.L.M., Muraru D., Edvardsen T., Cosyns B., Popescu B.A., Klein A., Marwick T.H. (2025). The Role of Multi-Modality Imaging for the Assessment of Left Atrium and Left Atrial Appendage: A Clinical Consensus Statement of the European Association of Cardiovascular Imaging (EACVI), European Heart Rhythm Association (EHRA) of the European Society of Cardiology (ESC). Eur. Heart J.-Cardiovasc. Imaging.

[15] Garcia-Pavia P., Rapezzi C., Adler Y., Arad M., Basso C., Brucato A., Burazor I., Caforio A.L.P., Damy T., Eriksson U. (2021). Diagnosis and Treatment of Cardiac Amyloidosis: A Position Statement of the ESC Working Group on Myocardial and Pericardial Diseases. Eur. Heart J..

[16] Ruberg F.L., Grogan M., Hanna M., Kelly J.W., Maurer M.S. (2019). Transthyretin Amyloid Cardiomyopathy: JACC State-of-the-Art Review. J. Am. Coll. Cardiol..

[17] Witteles R., Jefferies J.L., Kapa S., Cappelli F., Sultan M.B., Gundapaneni B., Davis M.K., Garcia-Pavia P. (2024). Atrial Fibrillation as a Prognostic Factor for All-Cause Mortality in Patients with Transthyretin Amyloid Cardiomyopathy. JACC CardioOncol..

[18] Ullah W., Ruge M., Hajduczok A.G., Kochar K., Frisch D.R., Pavri B.B., Alvarez R., Rajapreyar I.N., Brailovsky Y. (2023). Adverse Outcomes of Atrial Fibrillation Ablation in Heart Failure Patients with and without Cardiac Amyloidosis: A Nationwide Readmissions Database Analysis (2015–2019). Eur. Heart J. Open.

[19] Donnellan E., Wazni O.M., Hanna M., Elshazly M.B., Puri R., Saliba W., Kanj M., Vakamudi S., Patel D.R., Baranowski B. (2020). Atrial Fibrillation in Transthyretin Cardiac Amyloidosis: Predictors, Prevalence, and Efficacy of Rhythm Control Strategies. JACC Clin. Electrophysiol..

[20] Bukhari S., Barakat A.F., Eisele Y.S., Nieves R., Jain S., Saba S., Follansbee W.P., Brownell A., Soman P. (2021). Prevalence of Atrial Fibrillation and Thromboembolic Risk in Wild-Type Transthyretin Amyloid Cardiomyopathy. Circulation.

[21] Mints Y.Y., Doros G., Berk J.L., Connors L.H., Ruberg F.L. (2018). Features of Atrial Fibrillation in Wild-Type Transthyretin Cardiac Amyloidosis: A Systematic Review and Clinical Experience. ESC Heart Fail..

[22] Papathanasiou M., Jakstaite A.-M., Oubari S., Siebermair J., Wakili R., Hoffmann J., Carpinteiro A., Hagenacker T., Thimm A., Rischpler C. (2022). Clinical Features and Predictors of Atrial Fibrillation in Patients with Light-Chain or Transthyretin Cardiac Amyloidosis. ESC Heart Fail..

[23] Chan N., Brailovsky Y., Teruya S., Mirabal A., Weinsaft A.Y., De Los Santos J., Guadalupe S., Jimenez M., Helmke S., Cuomo M. (2026). Atrial Fibrillation/Flutter in Transthyretin Cardiac Amyloidosis. JACC Adv..

[24] Longhi S., Quarta C.C., Milandri A., Lorenzini M., Gagliardi C., Manuzzi L., Bacchi-Reggiani M.L., Leone O., Ferlini A., Russo A. (2015). Atrial Fibrillation in Amyloidotic Cardiomyopathy: Prevalence, Incidence, Risk Factors and Prognostic Role. Amyloid.

[25] Sanchis K., Cariou E., Colombat M., Ribes D., Huart A., Cintas P., Fournier P., Rollin A., Carrié D., Galinier M. (2019). Atrial Fibrillation and Subtype of Atrial Fibrillation in Cardiac Amyloidosis: Clinical and Echocardiographic Features, Impact on Mortality. Amyloid.

[26] Vergaro G., Aimo A., Rapezzi C., Castiglione V., Fabiani I., Pucci A., Buda G., Passino C., Lupón J., Bayes-Genis A. (2022). Atrial Amyloidosis: Mechanisms and Clinical Manifestations. Eur. J. Heart Fail..

[27] Bazoukis G., Saplaouras A., Efthymiou P., Yiannikourides A., Liu T., Sfairopoulos D., Korantzopoulos P., Varrias D., Letsas K.P., Thomopoulos C. (2024). Atrial Fibrillation in the Setting of Cardiac Amyloidosis—A Review of the Literature. J. Cardiol..

[28] Shinzato K., Takahashi Y., Yamaguchi T., Otsubo T., Nakashima K., Yoshioka G., Yokoi K., Tsuruta K., Osako R., Shichida S. (2025). Atrial Amyloidosis Identified by Biopsy in Atrial Fibrillation: Prevalence and Clinical Presentation. Eur. Heart J..

[29] Dal Passo B., Arzenton M., Cantone A., Fabbri G., Bonini J., Guidi Colombi G., Melpignano A., Marchini F., Izzo C., Pavasini R. (2025). Total Atrial Conduction Time Predicts New-Onset Atrial Fibrillation in Patients with Transthyretin Amyloid Cardiomyopathy. Europace.

[30] Sinigiani G., De Michieli L., Porcari A., Zocchi C., Sorella A., Mazzoni C., Bisaccia G., De Luca A., Di Bella G., Gregori D. (2024). Atrial Electrofunctional Predictors of Incident Atrial Fibrillation in Cardiac Amyloidosis. Heart Rhythm..

[31] Nagueh S.F. (2023). Left Atrial Function in Cardiac Amyloidosis. JACC Cardiovasc. Imaging.

[32] Cameli M., Caputo M., Mondillo S., Ballo P., Palmerini E., Lisi M., Marino E., Galderisi M. (2009). Feasibility and Reference Values of Left Atrial Longitudinal Strain Imaging by Two-Dimensional Speckle Tracking. Cardiovasc. Ultrasound.

[33] Meucci M.C., Lillo R., Mango F., Marsilia M., Iannaccone G., Tusa F., Luigetti M., Biagini E., Massetti M., Lanza G.A. (2024). Left Atrial Structural and Functional Remodelling in Fabry Disease and Cardiac Amyloidosis: A Comparative Analysis. Int. J. Cardiol..

[34] Monte I.P., Faro D.C., Trimarchi G., de Gaetano F., Campisi M., Losi V., Teresi L., Di Bella G., Tamburino C., de Gregorio C. (2023). Left Atrial Strain Imaging by Speckle Tracking Echocardiography: The Supportive Diagnostic Value in Cardiac Amyloidosis and Hypertrophic Cardiomyopathy. J. Cardiovasc. Dev. Dis..

[35] Rausch K., Scalia G.M., Sato K., Edwards N., Lam A.K.-Y., Platts D.G., Chan J. (2021). Left Atrial Strain Imaging Differentiates Cardiac Amyloidosis and Hypertensive Heart Disease. Int. J. Cardiovasc. Imaging.

[36] Minamisawa M., Inciardi R.M., Claggett B., Cuddy S.A.M., Quarta C.C., Shah A.M., Dorbala S., Falk R.H., Matsushita K., Kitzman D.W. (2021). Left Atrial Structure and Function of the Amyloidogenic V122I Transthyretin Variant in Elderly African Americans. Eur. J. Heart Fail..

[37] Akintoye E., Majid M., Klein A.L., Hanna M. (2023). Prognostic Utility of Left Atrial Strain to Predict Thrombotic Events and Mortality in Amyloid Cardiomyopathy. JACC Cardiovasc. Imaging.

[38] Porcari A., Passo B.D., Venneri L., Aimo A., Sezer Z.I., Bandera F., Razvi Y., Sheikh A., Mansell J., Rauf M.U. (2026). Atrial Mechanical Contraction Predicts Cerebrovascular Risk in Patients with Transthyretin Amyloid Cardiomyopathy and Sinus Rhythm. J. Am. Coll. Cardiol..

[39] Gao X., Xiao W., Ji L., Li H., Zou A., Miao Z., Zhang X., Yu S. (2025). Prognostic Implications of the Left Atrial Stiffness Index in Patients with Cardiac Amyloidosis. Int. J. Cardiol..

[40] Ferkh A., Geenty P., Stefani L., Emerson P., Pham J., Byth K., Boyd A.C., Richards D., Taylor M.S., Kwok F. (2024). Diagnostic and Prognostic Value of the Left Atrial Myopathy Evaluation in Cardiac Amyloidosis Using Echocardiography. ESC Heart Fail..

[41] Pepi M., Evangelista A., Nihoyannopoulos P., Flachskampf F.A., Athanassopoulos G., Colonna P., Habib G., Ringelstein E.B., Sicari R., Zamorano J.L. (2010). Recommendations for Echocardiography Use in the Diagnosis and Management of Cardiac Sources of Embolism: European Association of Echocardiography (EAE) (a Registered Branch of the ESC). Eur. J. Echocardiogr..

[42] Martinez-Naharro A., Gonzalez-Lopez E., Corovic A., Mirelis J.G., Baksi A.J., Moon J.C., Garcia-Pavia P., Gillmore J.D., Hawkins P.N., Fontana M. (2019). High Prevalence of Intracardiac Thrombi in Cardiac Amyloidosis. J. Am. Coll. Cardiol..

[43] Garcia-Pavia P., Gonzalez-Lopez E., Anderson L.J., Cappelli F., Damy T., Fontana M., Gonzalez-Costello J., Jurcut R., Lairez O., Van Der Meer P. (2026). Non-Amyloid Specific Treatment for Transthyretin Cardiac Amyloidosis: A Clinical Consensus Statement of the ESC Heart Failure Association. Eur. Heart J..

[44] Kittleson M.M., Ruberg F.L., Ambardekar A.V., Brannagan T.H., Cheng R.K., Clarke J.O., Dember L.M., Frantz J.G., Hershberger R.E., Maurer M.S. (2023). 2023 ACC Expert Consensus Decision Pathway on Comprehensive Multidisciplinary Care for the Patient with Cardiac Amyloidosis. J. Am. Coll. Cardiol..

[45] Poledniczek M., Kronberger C., Gregshammer B., List L., Willixhofer R., Ermolaev N., Duca F., Rettl R., Binder C., Camuz Ligios L. (2025). Left Atrial Appendage Thrombi despite Oral Anticoagulation in Transthyretin Amyloid Cardiomyopathy Patients Undergoing Electrical Cardioversion for Atrial Fibrillation or−Flutter. IJC Heart Vasc..

[46] El-Am E.A., Dispenzieri A., Melduni R.M., Ammash N.M., White R.D., Hodge D.O., Noseworthy P.A., Lin G., Pislaru S.V., Egbe A.C. (2019). Direct Current Cardioversion of Atrial Arrhythmias in Adults with Cardiac Amyloidosis. J. Am. Coll. Cardiol..

[47] Itzhaki Ben Zadok O., Cuddy S.A.M., Gaggin H.K., Clerc O.F., Vijayakumar S., Dorbala S., Falk R.H. (2024). Safety of Direct Current Cardioversion Without Routine Transesophageal Echocardiography in Patients with Cardiac Amyloidosis. J. Am. Coll. Cardiol..

[48] Ibrahim R., Colapietro L.D.L.B., Pham H.N., Abdelnabi M., Pietri M.P., Steidley D.E., Sorajja D., Arsanjani R., Rosenthal J., Ayoub C. (2025). Pre-Cardioversion Transesophageal Echocardiography in Patients with Transthyretin Cardiac Amyloidosis and Atrial Fibrillation. Am. J. Cardiol..

[49] Aimo A., Vergaro G., Panichella G., Castiglione V., Emdin M. (2025). Transesophageal Echocardiography Before Elective Direct Current Cardioversion in Cardiac Amyloidosis. J. Am. Coll. Cardiol..

[50] Aquaro G.D., Morini S., Grigoratos C., Taborchi G., Di Bella G., Martone R., Vignini E., Emdin M., Olivotto I., Perfetto F. (2020). Electromechanical Dissociation of Left Atrium in Patients with Cardiac Amyloidosis by Magnetic Resonance: Prognostic and Clinical Correlates. Int. J. Cardiol. Heart Vasc..

[51] Mohty D., Boulogne C., Magne J., Varroud-Vial N., Martin S., Ettaif H., Fadel B.M., Bridoux F., Aboyans V., Damy T. (2016). Prognostic Value of Left Atrial Function in Systemic Light-Chain Amyloidosis: A Cardiac Magnetic Resonance Study. Eur. Heart J. Cardiovasc. Imaging.

[52] Pontecorboli G., Figueras I Ventura R.M., Carlosena A., Benito E., Prat-Gonzales S., Padeletti L., Mont L. (2016). Use of Delayed-Enhancement Magnetic Resonance Imaging for Fibrosis Detection in the Atria: A Review. Europace.

[53] Pradella M., Elbaz M.S.M., Lee D.C., Hong K., Passman R.S., Kholmovski E., Peters D.C., Baraboo J.J., Herzka D.A., Nezafat R. (2025). A Comprehensive Evaluation of the Left Atrium Using Cardiovascular Magnetic Resonance. J. Cardiovasc. Magn. Reson..

[54] Malcolme-Lawes L.C., Juli C., Karim R., Bai W., Quest R., Lim P.B., Jamil-Copley S., Kojodjojo P., Ariff B., Davies D.W. (2013). Automated Analysis of Atrial Late Gadolinium Enhancement Imaging That Correlates with Endocardial Voltage and Clinical Outcomes: A 2-Center Study. Heart Rhythm..

[55] Marrouche N.F., Wilber D., Hindricks G., Jais P., Akoum N., Marchlinski F., Kholmovski E., Burgon N., Hu N., Mont L. (2014). Association of Atrial Tissue Fibrosis Identified by Delayed Enhancement MRI and Atrial Fibrillation Catheter Ablation: The DECAAF Study. JAMA.

[56] Kwong R.Y., Heydari B., Abbasi S., Steel K., Al-Mallah M., Wu H., Falk R.H. (2015). Characterization of Cardiac Amyloidosis by Atrial Late Gadolinium Enhancement Using Contrast-Enhanced Cardiac Magnetic Resonance Imaging and Correlation with Left Atrial Conduit and Contractile Function. Am. J. Cardiol..

[57] Kitkungvan D., Nabi F., Ghosn M.G., Dave A.S., Quinones M., Zoghbi W.A., Valderrabano M., Shah D.J. (2016). Detection of LA and LAA Thrombus by CMR in Patients Referred for Pulmonary Vein Isolation. JACC Cardiovasc. Imaging.

[58] Chen J., Zhang H., Zhu D., Wang Y., Byanju S., Liao M. (2019). Cardiac MRI for Detecting Left Atrial/Left Atrial Appendage Thrombus in Patients with Atrial Fibrillation: Meta-Analysis and Systematic Review. Herz.

[59] Solsona-Caravaca J., Giustiniani A., Ródenas-Alesina E., Galian-Gay L., Oliveró R., Valente F., Casas G., Teixidó-Turà G., Vallejo N., Fernández-Galera R. (2025). Comprehensive Assessment of Left Atrial Function: The Emerging Role of Cardiac Magnetic Resonance Feature Tracking. J. Cardiovasc. Dev. Dis..

[60] Sciacca V., Eckstein J., Körperich H., Fink T., Bergau L., El Hamriti M., Imnadze G., Guckel D., Fox H., Gerçek M. (2022). Magnetic-Resonance-Imaging-Based Left Atrial Strain and Left Atrial Strain Rate as Diagnostic Parameters in Cardiac Amyloidosis. J. Clin. Med..

[61] Tan Z., Yang Y., Wu X., Li S., Li L., Zhong L., Lin Q., Fei H., Liao P., Wang W. (2022). Left Atrial Remodeling and the Prognostic Value of Feature Tracking Derived Left Atrial Strain in Patients with Light-Chain Amyloidosis: A Cardiovascular Magnetic Resonance Study. Int. J. Cardiovasc. Imaging.

[62] García Méndez F.M., Fonseca-Marrero C., Sosa González I., Pérez Barreda A., Roldán-Nofuentes J.A., Ávila Cabreja J.A. (2026). Prognostic Value of Left Atrial Function in Cardiac Amyloidosis: A Systematic Review and Meta-analysis. Int. J. Cardiovasc. Imaging.

[63] Spartera M., Pessoa-Amorim G., Stracquadanio A., Von Ende A., Fletcher A., Manley P., Neubauer S., Ferreira V.M., Casadei B., Hess A.T. (2021). Left Atrial 4D Flow Cardiovascular Magnetic Resonance: A Reproducibility Study in Sinus Rhythm and Atrial Fibrillation. J. Cardiovasc. Magn. Reson..

[64] Ginami G., Lòpez K., Mukherjee R.K., Neji R., Munoz C., Roujol S., Mountney P., Razavi R., Botnar R.M., Prieto C. (2019). Non-Contrast Enhanced Simultaneous 3D Whole-Heart Bright-Blood Pulmonary Veins Visualization and Black-Blood Quantification of Atrial Wall Thickness. Magn. Reson. Med..

[65] Beinart R., Khurram I.M., Liu S., Yarmohammadi H., Halperin H.R., Bluemke D.A., Gai N., van der Geest R.J., Lima J.A.C., Calkins H. (2013). Cardiac Magnetic Resonance T1 Mapping of Left Atrial Myocardium. Heart Rhythm..

[66] Gorrie N., Geenty P., Rye E., Sivasubramaniam V., Carroll A., McCaughan G., Thomas L., Fatkin D., Bart N. (2025). Atrial Cardiomyopathy in Cardiac Amyloidosis: Clinical Imaging and Manifestations. npj Cardiovasc. Health.

[67] Burke S.J., Aggarwala G., Stanford W., Mullan B., Thompson B., van Beek E.J.R. (2008). Preablation Assessment for the Left Atrium: Comparison of ECG-Gated Cardiac CT with Echocardiography. Acad. Radiol..

[68] Romero J., Husain S.A., Kelesidis I., Sanz J., Medina H.M., Garcia M.J. (2013). Detection of Left Atrial Appendage Thrombus by Cardiac Computed Tomography in Patients with Atrial Fibrillation: A Meta-Analysis. Circ. Cardiovasc. Imaging.

[69] Tubeeckx M.R.L., De Keulenaer G.W., Heidbuchel H., Segers V.F.M. (2024). Pathophysiology and Clinical Relevance of Atrial Myopathy. Basic Res. Cardiol..

[70] Abazid R.M., Romsa J.G., Warrington J.C., Stodilka R.Z., Davey R.A., De S., Laidley D.L., Vezina W.C., Akincioglu C. (2022). Tc-99m Pyrophosphate Left Atrial Uptake in Patients with Atrial Fibrillation and Cardiac Amyloidosis. J. Nucl. Cardiol..

[71] Wall J.S., Martin E.B., Lands R., Ramchandren R., Stuckey A., Heidel R.E., Whittle B., Powell D., Richey T., Williams A.D. (2023). Cardiac Amyloid Detection by PET/CT Imaging of Iodine (124I) Evuzamitide (124I-P5+14): A Phase 1/2 Study. JACC Cardiovasc. Imaging.

[72] Vilches S., Fontana M., Gonzalez-Lopez E., Mitrani L., Saturi G., Renju M., Griffin J.M., Caponetti A., Gnanasampanthan S., De Los Santos J. (2022). Systemic Embolism in Amyloid Transthyretin Cardiomyopathy. Eur. J. Heart Fail..

[73] Donnellan E., Elshazly M.B., Vakamudi S., Wazni O.M., Cohen J.A., Kanj M., Hanna M., Baranowski B., Saliba W., Jaber W. (2019). No Association Between CHADS-VASc Score and Left Atrial Appendage Thrombus in Patients with Transthyretin Amyloidosis. JACC Clin. Electrophysiol..

[74] Giancaterino S., Urey M.A., Darden D., Hsu J.C. (2020). Management of Arrhythmias in Cardiac Amyloidosis. JACC Clin. Electrophysiol..

[75] Arbelo E., Protonotarios A., Gimeno J.R., Arbustini E., Barriales-Villa R., Basso C., Bezzina C.R., Biagini E., Blom N.A., de Boer R.A. (2023). 2023 ESC Guidelines for the Management of Cardiomyopathies. Eur. Heart J..

[76] Tana M., Tana C., Rossi D., Mantini C., Gallina S., Ricci F., Porreca E. (2024). Thromboembolic and Bleeding Risk in Cardiac Amyloidosis. J. Thromb. Haemost..

[77] Nong Q., Liang S., Zhu W., Chen Y., Zhang T. (2025). Direct Oral Anticoagulants vs. Vitamin K Antagonists for Atrial Fibrillation in Cardiac Amyloidosis: A Systematic Review and Meta-Analysis. Rev. Cardiovasc. Med..

[78] Amat-Santos I.J., Delgado-Arana J.R., Cruz-González I., Gutiérrez H., García-Bolao I., Millán X., Tirado-Conte G., Ruiz-Nodar J.M., Mohandes M., Palazuelos J. (2023). Cardiac Amyloidosis and Left Atrial Appendage Closure. The CAMYLAAC Study. Rev. Esp. Cardiol. (Engl. Ed.).

[79] Guenancia C., Lequeux B., Amara W., Buiciuc O., Damy T., Defaye P., Duparc A., Eicher J.-C., Garcia R., Galand V. (2025). Management of Rhythm and Conduction Disorders in Cardiac Amyloidosis. JACC Adv..

[80] Falk R.H., Alexander K.M., Liao R., Dorbala S. (2016). AL (Light-Chain) Cardiac Amyloidosis: A Review of Diagnosis and Therapy. J. Am. Coll. Cardiol..

[81] Girvin Z.P., Sweat A.O., Kochav S.M., Maurer M.S., Dizon J., Wan E.Y., Biviano A., Garan H., Yarmohammadi H. (2023). Tafamidis and Incidence of Atrial Fibrillation in Transthyretin Amyloid Cardiomyopathy. JACC Clin. Electrophysiol..

[82] Cruz M.W. (2019). Tafamidis for Autonomic Neuropathy in Hereditary Transthyretin (ATTR) Amyloidosis: A Review. Clin. Auton. Res..

[83] Zeis T., Motairek I., Abadie B., Taigen T., Wazni O., Saliba W., Higgins A., Hanna M., Jaber W. (2026). Associations of Tafamidis with New Atrial Fibrillation Risk in Transthyretin Cardiomyopathy. JACC Clin. Electrophysiol..

[84] Linhart A., Germain D.P., Olivotto I., Akhtar M.M., Anastasakis A., Hughes D., Namdar M., Pieroni M., Hagège A., Cecchi F. (2020). An Expert Consensus Document on the Management of Cardiovascular Manifestations of Fabry Disease. Eur. J. Heart Fail..

[85] Roy A., Cumberland M.J., O’Shea C., Holmes A., Kalla M., Gehmlich K., Geberhiwot T., Steeds R.P. (2024). Arrhythmogenesis in Fabry Disease. Curr. Cardiol. Rep..

[86] Namdar M. (2016). Electrocardiographic Changes and Arrhythmia in Fabry Disease. Front. Cardiovasc. Med..

[87] Acharya D., Robertson P., Kay G.N., Jackson L., Warnock D.G., Plumb V.J., Tallaj J.A. (2012). Arrhythmias in Fabry Cardiomyopathy. Clin. Cardiol..

[88] Patel V., O’Mahony C., Hughes D., Rahman M.S., Coats C., Murphy E., Lachmann R., Mehta A., Elliott P.M. (2015). Clinical and Genetic Predictors of Major Cardiac Events in Patients with Anderson-Fabry Disease. Heart.

[89] Shah J.S., Hughes D.A., Sachdev B., Tome M., Ward D., Lee P., Mehta A.B., Elliott P.M. (2005). Prevalence and Clinical Significance of Cardiac Arrhythmia in Anderson-Fabry Disease. Am. J. Cardiol..

[90] Pieroni M., Namdar M., Olivotto I., Desnick R.J. (2024). Anderson-Fabry Disease Management: Role of the Cardiologist. Eur. Heart J..

[91] Fan J.-L., Su B., Zhao X., Zhou B.-Y., Ma C.-S., Wang H.-P., Hu S.-D., Zhou Y.-F., Ju Y.-J., Wang M.-H. (2020). Correlation of Left Atrial Strain with Left Ventricular End-Diastolic Pressure in Patients with Normal Left Ventricular Ejection Fraction. Int. J. Cardiovasc. Imaging.

[92] Birket M.J., Raibaud S., Lettieri M., Adamson A.D., Letang V., Cervello P., Redon N., Ret G., Viale S., Wang B. (2019). A Human Stem Cell Model of Fabry Disease Implicates LIMP-2 Accumulation in Cardiomyocyte Pathology. Stem Cell Rep..

[93] Cameli M., Pieroni M., Pastore M.C., Brucato A., Castelletti S., Crotti L., Dweck M., Frustaci A., Gimelli A., Klingel K. (2025). The Role of Cardiovascular Multimodality Imaging in the Evaluation of Anderson-Fabry Disease: From Early Diagnosis to Therapy Monitoring. Eur. Heart J. Cardiovasc. Imaging.

[94] Pichette M., Serri K., Pagé M., Di L.Z., Bichet D.G., Poulin F. (2017). Impaired Left Atrial Function in Fabry Disease: A Longitudinal Speckle-Tracking Echocardiography Study. J. Am. Soc. Echocardiogr..

[95] Stankowski K., Figliozzi S., Rojanathagoon T., Bampatsias D., Klettas D., Monti L., Bragato R., Masci P.-G., Francone M., Condorelli G. (2025). Imaging Predictors of Adverse Prognosis in Fabry Disease Cardiomyopathy: A Systematic Review and Meta-Analysis. Eur. J. Clin. Investig..

[96] Pieroni M., Moon J.C., Arbustini E., Barriales-Villa R., Camporeale A., Vujkovac A.C., Elliott P.M., Hagege A., Kuusisto J., Linhart A. (2021). Cardiac Involvement in Fabry Disease. J. Am. Coll. Cardiol..

[97] Halfmann M.C., Altmann S., Schoepf U.J., Reichardt C., Hennermann J.B., Kreitner K.-F., Kloeckner R., Hahn F., Dueber C., Varga-Szemes A. (2023). Left Atrial Strain Correlates with Severity of Cardiac Involvement in Anderson-Fabry Disease. Eur. Radiol..

[98] Bernardini A., Camporeale A., Pieroni M., Pieruzzi F., Figliozzi S., Lusardi P., Spada M., Mignani R., Burlina A., Carubbi F. (2020). Atrial Dysfunction Assessed by Cardiac Magnetic Resonance as an Early Marker of Fabry Cardiomyopathy. JACC Cardiovasc. Imaging.

[99] Cheepvasarach C., Gribble M., Ugander M., Vijapurapu R., Nordin S., Augusto J., Steeds R.P., Tchan M., Moon J.C., Pathan F. (2025). Left Atrial Strain Tracks Abnormal Ventricular Mechanics in Fabry Disease. Open Heart.

[100] Brodsky S.V., Nadasdy T., Rovin B.H., Satoskar A.A., Nadasdy G.M., Wu H.M., Bhatt U.Y., Hebert L.A. (2011). Warfarin-Related Nephropathy Occurs in Patients with and without Chronic Kidney Disease and Is Associated with an Increased Mortality Rate. Kidney Int..

[101] Reisin R.C., Romero C., Marchesoni C., Nápoli G., Kisinovsky I., Cáceres G., Sevlever G. (2011). Brain MRI Findings in Patients with Fabry Disease. J. Neurol. Sci..

[102] Ikeda K., Hirayama M., Hirota Y., Asa E., Seki J., Tanaka Y. (2008). Drug-Induced Phospholipidosis Is Caused by Blockade of Mannose 6-Phosphate Receptor-Mediated Targeting of Lysosomal Enzymes. Biochem. Biophys. Res. Commun..

[103] Fine N.M., Wang Y., Khan A. (2019). Acute Decompensated Heart Failure After Initiation of Amiodarone in a Patient with Anderson-Fabry Disease. Can. J. Cardiol..

[104] Ortiz A., Germain D.P., Desnick R.J., Politei J., Mauer M., Burlina A., Eng C., Hopkin R.J., Laney D., Linhart A. (2018). Fabry Disease Revisited: Management and Treatment Recommendations for Adult Patients. Mol. Genet. Metab..

[105] Germain D.P., Charrow J., Desnick R.J., Guffon N., Kempf J., Lachmann R.H., Lemay R., Linthorst G.E., Packman S., Scott C.R. (2015). Ten-Year Outcome of Enzyme Replacement Therapy with Agalsidase Beta in Patients with Fabry Disease. J. Med. Genet..

[106] Germain D.P., Hughes D.A., Nicholls K., Bichet D.G., Giugliani R., Wilcox W.R., Feliciani C., Shankar S.P., Ezgu F., Amartino H. (2016). Treatment of Fabry’s Disease with the Pharmacologic Chaperone Migalastat. N. Engl. J. Med..

[107] Lenders M., Nordbeck P., Kurschat C., Karabul N., Kaufeld J., Hennermann J.B., Patten M., Cybulla M., Müntze J., Üçeyler N. (2020). Treatment of Fabry’s Disease with Migalastat: Outcome from a Prospective Observational Multicenter Study (FAMOUS). Clin. Pharmacol. Ther..

[108] Roberts W.C., McAllister H.A., Ferrans V.J. (1977). Sarcoidosis of the Heart. A Clinicopathologic Study of 35 Necropsy Patients (Group 1) and Review of 78 Previously Described Necropsy Patients (Group 11). Am. J. Med..

[109] Birnie D.H., Kandolin R., Nery P.B., Kupari M. (2017). Cardiac Manifestations of Sarcoidosis: Diagnosis and Management. Eur. Heart J..

[110] Niemelä M., Uusitalo V., Pöyhönen P., Schildt J., Lehtonen J., Kupari M. (2022). Incidence and Predictors of Atrial Fibrillation in Cardiac Sarcoidosis: A Multimodality Imaging Study. JACC Cardiovasc. Imaging.

[111] Fujimoto Y., Matsue Y., Maeda D., Dotare T., Sunayama T., Iso T., Nakamura Y., Singh Y.S., Akama Y., Yoshioka K. (2023). Prevalence and Prognostic Value of Atrial Fibrillation in Patients with Cardiac Sarcoidosis. Eur. Heart J. Open.

[112] Sharma R., Kouranos V., Cooper L.T., Metra M., Ristic A., Heidecker B., Baksi J., Wicks E., Merino J.L., Klingel K. (2024). Management of Cardiac Sarcoidosis. Eur. Heart J..

[113] Cain M.A., Metzl M.D., Patel A.R., Addetia K., Spencer K.T., Sweiss N.J., Beshai J.F. (2014). Cardiac Sarcoidosis Detected by Late Gadolinium Enhancement and Prevalence of Atrial Arrhythmias. Am. J. Cardiol..

[114] Viles-Gonzalez J.F., Pastori L., Fischer A., Wisnivesky J.P., Goldman M.G., Mehta D. (2013). Supraventricular Arrhythmias in Patients with Cardiac Sarcoidosis Prevalence, Predictors, and Clinical Implications. Chest.

[115] Rosenfeld L.E., Chung M.K., Harding C.V., Spagnolo P., Grunewald J., Appelbaum J., Sauer W.H., Culver D.A., Joglar J.A., Lin B.A. (2021). Arrhythmias in Cardiac Sarcoidosis Bench to Bedside: A Case-Based Review. Circ. Arrhythm. Electrophysiol..

[116] Bhaskaran A., Kumar S., Kizana E., Thomas S.P., Chik W.W.B. (2018). Multimodality Imaging, Electrophysiologic, Electroanatomic, and Histopathologic Characterization of Atrial Sarcoidosis Presenting with Sinus Arrest and Reentrant Right Atrial Flutter. Hear. Case Rep..

[117] Fingrova Z., Havranek S., Ambroz D., Jansa P., Linhart A. (2019). The Left Atrial Substrate Plays a Significant Role in the Development of Complex Atrial Tachycardia in Patients with Precapillary Pulmonary Hypertension. BMC Cardiovasc. Disord..

[118] Namboodiri N., Stiles M.K., Young G.D., Sanders P. (2012). Electrophysiological Features of Atrial Flutter in Cardiac Sarcoidosis: A Report of Two Cases. Indian. Pacing Electrophysiol. J..

[119] Koike H., Yahagi K., Yuzawa H., Sato K., Asami M., Komiyama K., Tanaka J., Aoki J., Tanabe K., Ikeda T. (2019). Atypical Re-Entrant Circuit of Cavo-Tricuspid Isthmus-Dependent Atrial Flutter Due to an Atrial-Septal Conduction Disturbance in a Patient with Cardiac Sarcoidosis. JACC Clin. Electrophysiol..

[120] Enzan N., Ohtani K., Nagaoka K., Sakamoto I., Tsutsui H. (2019). Left Atrial Involvement of Cardiac Sarcoidosis Manifesting as Left Atrial Re-Entrant Tachycardia. Eur. Heart J. Cardiovasc. Imaging.

[121] Habibi M., Saad E., Okada D.R., Berger R.D., Gilotra N.A., Tandri H., Calkins H., Lima J.A., Rowe S., Chrispin J. (2021). Multimodality Imaging of Atrial Remodeling and Risk of Atrial Fibrillation in Patients with Cardiac Sarcoidosis. JACC Cardiovasc. Imaging.

[122] Yodogawa K., Fukushima Y., Ando T., Iwasaki Y.-K., Akiyama K., Kumita S.-I., Azuma A., Seino Y., Shimizu W. (2020). Prevalence of Atrial FDG Uptake and Association with Atrial Arrhythmias in Patients with Cardiac Sarcoidosis. Int. J. Cardiol..

[123] Rosenthal D.G., Fang C.D., Groh C.A., Nah G., Vittinghoff E., Dewland T.A., Marcus G.M. (2021). Association Between Sarcoidosis and Atrial Fibrillation Among Californians Using Medical Care. JACC Clin. Electrophysiol..

[124] Cameli P., Pastore M.C., Mandoli G.E., Vigna M., De Carli G., Bergantini L., d’Alessandro M., Ghionzoli N., Bargagli E., Cameli M. (2021). Strain Echocardiography Is a Promising Tool for the Prognostic Assessment of Sarcoidosis. Life.

[125] Jain H., Shahzad M., Ahsan M., Patel R., Singh J., Odat R.M., Goyal A., Kelkar R., Barve N., Farrukh H. (2025). Diagnostic Value of Comprehensive Echocardiographic Assessment Including Speckle-Tracking in Patients with Sarcoidosis Versus Healthy Controls: A Systematic Review and Meta-Analysis. Diagnostics.

[126] Tigen K., Sunbul M., Karaahmet T., Tasar O., Dundar C., Yalcinsoy M., Takir M., Akkaya E. (2015). Early Detection of Bi-Ventricular and Atrial Mechanical Dysfunction Using Two-Dimensional Speckle Tracking Echocardiography in Patients with Sarcoidosis. Lung.

[127] Van Gelder I.C., Rienstra M., Bunting K.V., Casado-Arroyo R., Caso V., Crijns H.J.G.M., De Potter T.J.R., Dwight J., Guasti L., Hanke T. (2024). 2024 ESC Guidelines for the Management of Atrial Fibrillation Developed in Collaboration with the European Association for Cardio-Thoracic Surgery (EACTS). Eur. Heart J..

[128] Yada H., Soejima K. (2019). Management of Arrhythmias Associated with Cardiac Sarcoidosis. Korean Circ. J..

[129] Weng W., Wiefels C., Chakrabarti S., Nery P.B., Celiker-Guler E., Healey J.S., Hruczkowski T.W., Quinn F.R., Promislow S., Medor M.C. (2020). Atrial Arrhythmias in Clinically Manifest Cardiac Sarcoidosis: Incidence, Burden, Predictors, and Outcomes. J. Am. Heart Assoc..

[130] Oraii A., Hanumanthu B.K.J., Petzl A., Liao T.-W., Afzalian A., Rodriguez-Queralto O., Chaumont C., Spears J., Markman T.M., Hyman M.C. (2025). Catheter Ablation of Atrial Fibrillation in Patients with Cardiac Amyloidosis and Sarcoidosis: Procedural Findings and Outcomes. Europace.

[131] Mohsen A. (2011). The Anti-Arrhythmic Effects of Prednisone in Patients with Sarcoidosis. Acta Cardiol..

[132] Golwala H., Dernaika T. (2015). Atrial Fibrillation as the Initial Clinical Manifestation of Cardiac Sarcoidosis: A Case Report and Review of the Literature. J. Cardiovasc. Med..

[133] Hasegawa K., Kaseno K., Aiki T., Tada H. (2018). Left Atrial Sarcoidosis as a Substrate for Peri-Mitral Atrial Flutter: An Unusual, Underlying Atrial Disease. Eur. Heart J..

[134] Malagù M., Marchini F., Fiorio A., Sirugo P., Clò S., Mari E., Gamberini M.R., Rapezzi C., Bertini M. (2022). Atrial Fibrillation in β-Thalassemia: Overview of Mechanism, Significance and Clinical Management. Biology.

[135] Jackson I., Oyenuga M., Balogun O., Oyenuga A., Etuk A., Jackson N. (2022). Retrospective Analyses of Factors Influencing Arrhythmias and the Impact of Arrhythmias on Inpatient Outcomes among Hospitalized Patients with Hemochromatosis. Int. J. Cardiol..

[136] Shizukuda Y., Tripodi D.J., Zalos G., Bolan C.D., Yau Y.-Y., Leitman S.F., Waclawiw M.A., Rosing D.R. (2012). Incidence of Cardiac Arrhythmias in Asymptomatic Hereditary Hemochromatosis Subjects with C282Y Homozygosity. Am. J. Cardiol..

[137] Kirk P., Roughton M., Porter J.B., Walker J.M., Tanner M.A., Patel J., Wu D., Taylor J., Westwood M.A., Anderson L.J. (2009). Cardiac T2* Magnetic Resonance for Prediction of Cardiac Complications in Thalassemia Major. Circulation.

[138] Kremastinos D.T., Farmakis D. (2011). Iron Overload Cardiomyopathy in Clinical Practice. Circulation.

[139] Gujja P., Rosing D.R., Tripodi D.J., Shizukuda Y. (2010). Iron Overload Cardiomyopathy: Better Understanding of an Increasing Disorder. J. Am. Coll. Cardiol..

[140] Rose R.A., Sellan M., Simpson J.A., Izaddoustdar F., Cifelli C., Panama B.K., Davis M., Zhao D., Markhani M., Murphy G.G. (2011). Iron Overload Decreases CaV1.3-Dependent L-Type Ca2^+^ Currents Leading to Bradycardia, Altered Electrical Conduction, and Atrial Fibrillation. Circ. Arrhythm. Electrophysiol..

[141] Wood J.C. (2008). Cardiac Iron across Different Transfusion-Dependent Diseases. Blood Rev..

[142] Kostopoulou A.G., Tsiapras D.P., Chaidaroglou A.S., De Giannis D.E., Farmakis D., Kremastinos D.T. (2014). The Pathophysiological Relationship and Clinical Significance of Left Atrial Function and Left Ventricular Diastolic Dysfunction in β-Thalassemia Major. Am. J. Hematol..

[143] Palka P., Macdonald G., Lange A., Burstow D.J. (2002). The Role of Doppler Left Ventricular Filling Indexes and Doppler Tissue Echocardiography in the Assessment of Cardiac Involvement in Hereditary Hemochromatosis. J. Am. Soc. Echocardiogr..

[144] Saad A.K., Aladio J.M., Yamasato F., Volberg V.I., Gonzalez Ballerga E., Sordá J.A., Daruich J., Perez de la Hoz R.A. (2022). Analysis of The Left Atrial Function Using Two-Dimensional Strain in Patients with Recent Diagnosis of Hereditary Hemochromatosis. Curr. Probl. Cardiol..

[145] Marchini F., Mele M., Marchetti E., Rotondo L., Frascaro F., Malagù M., Pavasini R., Tonet E., Serenelli M., Cossu A. (2026). Assessing Cardiac Mechanical Dysfunction in Transfusion-Dependent β-Thalassemia with History of Atrial Fibrillation: The Role of Speckle Tracking Echocardiography. Echocardiography.

[146] Meloni A., Saba L., Positano V., Pistoia L., Porcu M., Massei F., Sanna P.M.G., Longo F., Giovangrossi P., Argento C. (2024). Left Atrial Strain in Patients with β-Thalassemia Major: A Cross-Sectional CMR Study. Eur. Radiol..

[147] Origa R., Danjou F., Cossa S., Matta G., Bina P., Dessì C., Defraia E., Foschini M.L., Leoni G., Morittu M. (2013). Impact of Heart Magnetic Resonance Imaging on Chelation Choices, Compliance with Treatment and Risk of Heart Disease in Patients with Thalassaemia Major. Br. J. Haematol..

[148] Pennell D.J., Udelson J.E., Arai A.E., Bozkurt B., Cohen A.R., Galanello R., Hoffman T.M., Kiernan M.S., Lerakis S., Piga A. (2013). Cardiovascular Function and Treatment in β-Thalassemia Major: A Consensus Statement from the American Heart Association. Circulation.

[149] Oudit G.Y., Sun H., Trivieri M.G., Koch S.E., Dawood F., Ackerley C., Yazdanpanah M., Wilson G.J., Schwartz A., Liu P.P. (2003). L-Type Ca2^+^ Channels Provide a Major Pathway for Iron Entry into Cardiomyocytes in Iron-Overload Cardiomyopathy. Nat. Med..

